# Drug-delivery strategies using biomaterials in the field of nerve regeneration

**DOI:** 10.4103/NRR.NRR-D-25-00027

**Published:** 2025-06-19

**Authors:** Linbin Xu, Chao Zhou, Xu Wang, Cunyi Fan

**Affiliations:** 1 Department of Orthopedics, Shanghai Sixth People’s Hospital Affiliated to Shanghai Jiao Tong University School of Medicine, Shanghai, China; 2 National Center for Orthopedics, Shanghai, China; 3 Shanghai Engineering Research Center for Orthopedic Material Innovation and Tissue Regeneration, Shanghai, China; 4 National Center for Translational Medicine (Shanghai) SHU Branch, Shanghai, China

**Keywords:** biomaterials, clinical trial, drug, drug-delivery strategy, drug-loading strategy, drug-release strategy, nerve regeneration, peripheral nerve, RNA, tissue engineering

## Abstract

Neural injuries can cause considerable functional impairments, and both central and peripheral nervous systems have limited regenerative capacity. The existing conventional pharmacological treatments in clinical practice show poor targeting, rapid drug clearance from the circulatory system, and low therapeutic efficiency. Therefore, in this review, we have first described the mechanisms underlying nerve regeneration, characterized the biomaterials used for drug delivery to facilitate nerve regeneration, and highlighted the functionalization strategies used for such drug-delivery systems. These systems mainly use natural and synthetic polymers, inorganic materials, and hybrid systems with advanced drug-delivery abilities, including nanoparticles, hydrogels, and scaffold-based systems. Then, we focused on comparing the types of drug-delivery systems for neural regeneration as well as the mechanisms and challenges associated with targeted delivery of drugs to facilitate neural regeneration. Finally, we have summarized the clinical application research and limitations of targeted delivery of these drugs. These biomaterials and drug-delivery systems can provide mechanical support, sustained release of bioactive molecules, and enhanced intercellular contact, ultimately reducing cell apoptosis and enhancing functional recovery. Nevertheless, immune reactions, degradation regulation, and clinical translations remain major unresolved challenges. Future studies should focus on optimizing biomaterial properties, refining delivery precision, and overcoming translational barriers to advance these technologies toward clinical applications.

## Introduction

The central nervous system (CNS) and the peripheral nervous system (PNS) are the most complex communication systems in the human body. Because of the poor regenerative capacity of neurons and axons, traumatic injuries, inflammation, tumors, metabolic disorders such as diabetes, and neurodegenerative diseases such as Alzheimer’s disease often cause irreversible damage and loss of function in the peripheral nerves, spinal cord, and brain (Wüthrich et al., 2020; Varadarajan et al., 2022; De Virgiliis et al., 2023). Traditional drug therapy involves systemic administration (oral or intravenous), wherein the drug tends to accumulate in non-target organs, causing side effects, and is quickly eliminated, preventing the drug from reaching the target tissue. More recently, substantial advancements in biomaterials and drug-delivery systems have been made to tackle these challenges. Through well-designed drug-delivery strategies, regenerative drugs or growth factors can be protected from degradation or denaturation, ensuring their stability and sustained release at specific sites. This approach can facilitate nerve repair and reduce inflammatory responses in addition to yielding enhanced therapeutic efficacy.

The biomaterials employed in drug delivery that are covered in this review include natural polymers, synthetic polymers, and inorganic minerals such as collagen (Geiger et al., 2003), silk (Li et al., 2006), chitosan (Yilgor et al., 2009), fibrin (Spicer and Mikos, 2010), hyaluronic acid (Prestwich, 2011), alginate (Kolambkar et al., 2011), gelatin (Wang et al., 2012), and glycosaminoglycans (GAGs) (Miller et al., 2014). Because of their biocompatibility and biodegradability, these biomaterials have received wide attention. Furthermore, the synthetic polymers that have been extensively studied due to their tunable physicochemical properties include polylactic acid (PLA), polyethylene glycol (PEG) (Burdick et al., 2002), poly(lactic-co-glycolic acid) (PLGA) (Makadia and Siegel, 2011), poly(propylene fumarate) (PPF) (Kempen et al., 2008), polycaprolactone (PCL) (Dash and Konkimalla, 2012), polyethyleneimine (PEI) (Fattahi et al., 2024), and conducting polymers (CPs) (Yi et al., 2023). Moreover, inorganic materials such as mesoporous silica (MS) (Xu et al., 2023a), gold (Han et al., 2023), hydroxyapatite (Mondal et al., 2023), calcium phosphate (CaP) (Khalifehzadeh and Arami, 2020), zinc oxide (ZnO) (Król et al., 2017), and graphene (Song et al., 2020) have also demonstrated unique advantages in drug-delivery applications. Various biomaterials formed through these substances include hydrogels, microspheres, and nanoparticles. In addition, technological improvements in drug formulations have also rendered cell-based and cell-derived exosome-based targeted drug-delivery systems as promising modalities for nerve tissue regeneration with high immunogenicity, intrinsic mutation rates, and the potential of allowing targeted delivery (Wu et al., 2019; Li et al., 2023f). Several studies have validated the efficacy of extracellular vesicles in experimental animals (Zhang et al., 2021a; Chen et al., 2022; Zhu et al., 2023a; Lian et al., 2024; Song et al., 2024; Wang et al., 2024). Moreover, lipid carriers have gained substantial attention in recent years as efficient materials for the delivery of nucleic acid–based therapeutics and small-molecule drugs (Mishra et al., 2018). However, clinical trials of these drug-delivery systems are still in the preliminary stage, and literature on their long-term stability and translational effectiveness is relatively limited.

At present, research on biomaterials and drug-delivery strategies in the field of neuroregenerative drug delivery is still limited by some factors. Although substantial progress has been made in research and development of related technologies and materials, comprehensive and systematic research is still insufficient. Therefore, this review will systematically summarize and analyze the core technologies, challenges, and future development directions in this field. The review will first explore the biological basis of nerve regeneration, elucidating the mechanisms of nerve injury and the challenges associated with treating it. Next, we provide a detailed introduction to the classification and characteristics of various biomaterials used to prepare drug-delivery systems, discussing their roles in drug delivery. Subsequently, we describe the different ways drugs are loaded onto delivery systems, as well as the types, mechanisms, challenges, and clinical applications of the different neuroregenerative drugs used in such systems. Finally, we provide some new insights into the future development of neural tissue regeneration in the field of medicine.

## Search Strategies

To ensure that this review encompassed the latest research advancements in neuroregenerative drug-delivery systems, a systematic literature search strategy was employed. We conducted searches across multiple authoritative databases, including PubMed, Web of Science, and Google Scholar, which extensively cover high-quality research in the fields of medicine, bioengineering, and materials science. Additionally, ClinicalTrials.gov and the Chinese Clinical Trial Registry were searched to obtain information on ongoing and completed clinical trials, with Google Scholar serving as a supplementary resource to ensure comprehensive coverage. During the search process, we focused on core topics such as nerve regeneration, drug-delivery systems, nanotechnology, gene therapy, and biomaterials, formulating a series of refined search strategies. To enhance the accuracy of retrieval, we implemented specific screening methods to ensure that the search results comprehensively encompassed all key research findings while minimizing irrelevant information. For literature selection, priority was assigned to studies published within the last 3 years, with emphasis on high-impact journal articles that had undergone peer review. However, foundational studies with significant contributions to the field were also included selectively to provide a more comprehensive perspective on the developmental trajectory. Simultaneously, to maintain the scientific rigor and reliability of this review, we excluded studies limited to *in vitro* experiments, research with incomplete data, and publications that lacked peer review.

## Biological Basis of Nerve Regeneration

### Structure and functions of the nervous system and mechanisms underlying nerve injury

The human and mammalian nervous system is highly complex, consisting of the CNS and the PNS. The CNS, which consists of the brain and spinal cord, processes information and regulates bodily functions. The cerebral cortex handles high-level cognition; the cerebellum coordinates movement; and the brainstem controls vital functions (Yang et al., 2024a; Fukushi et al., 2025). The spinal cord transmits signals and manages reflex responses. The PNS, which includes the cranial and spinal nerves, connects the CNS to the body, with afferent nerves transmitting sensory input and efferent nerves driving responses (Mueller et al., 2024). The autonomic nervous system, a subset of the PNS, consists of the sympathetic and parasympathetic systems, which regulate involuntary functions (Arslan and Ünal Çevik, 2022; Mueller et al., 2024). At the cellular level, neurons, which are composed of dendrites, cytosol, and axons, form the fundamental units of the nervous system, enabling signal transmission and response coordination (Jessen and Mirsky, 2008; Bott and Winckler, 2020).

Nerve injuries can be categorized as central and peripheral nerve injuries, of which peripheral nerve injuries are often caused by factors such as traffic accidents, natural disasters, war injuries, disease complications, and extreme sports accidents (Li et al., 2022; Hao et al., 2024). Most injuries to neurons in the CNS are irreversible due to the neurons’ limited regenerative capacity, which has been attributed to the slow clearance process and unfavorable microenvironmental factors along with the inhibitory effects of oligodendrocytes on neuronal regeneration (Tsujioka and Yamashita, 2021). In contrast, peripheral neurons possess regenerative potential. Thus, the mechanisms underlying nerve injury can be investigated in terms of the type of nerve injury.

### Biological processes in nerve regeneration

**Figure 1:**
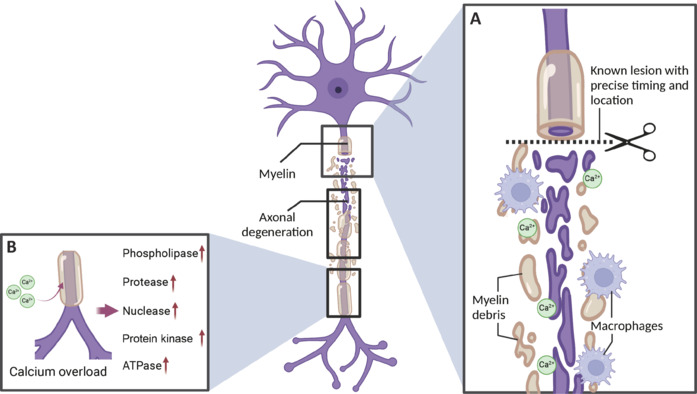
Wallerian degeneration: mechanisms involving calcium overload and enzyme activation. (A) A specific lesion is introduced in the axon, leading to the breakdown of myelin and axonal degeneration. (B) Calcium overload triggers enzymatic cascades, leading to axonal degeneration. Created with BioRender.com.

A series of events are triggered after nerve injury. The early stages of nerve injury are characterized by rapid weakening or loss of nerve fiber conduction, which results in polarity changes in nerve function. Peripheral nerve injury due to trauma triggers a cascade of cellular and molecular events, beginning with Wallerian degeneration across two phases: early (**~**5 days) and late (**~**5–14 days) (**Figure 1**; Fissel and Farah, 2021). In the early phase, the injured nerve is detached from the distally located nerve trunk and undergoes cellular changes such as axonal breaks, in addition to showing alterations in the membrane potential of the nerve cell and abnormalities in the functioning of ion channels, which impede normal conduction of nerve impulses. Calcium overload triggers enzymatic cascades, leading to axonal degeneration. The influx of Ca^2+^ into the distal nerve stumps marks the onset of Wallerian degeneration at the molecular level (Fissel and Farah, 2021). A significant increase in the intracellular Ca^2+^ concentration causes rapid activation of calpain, which is extremely sensitive to Ca^2+^ concentration and acts on cytoskeletal proteins that maintain cell structure and function. Additionally, the upregulation of phospholipase, protease, nuclease, protein kinase, and ATPase expression contributes to cellular breakdown and degradation processes. Simultaneously, edema and inflammation immediately appear at the site of injury, and the infiltration of inflammatory cells releases a variety of inflammatory mediators, which further exacerbate the neurological dysfunction.

With time, the recovery process following nerve injury gradually enters the regeneration phase. Nerve regeneration primarily occurs in the later stages of injury, and this process involves axonal regeneration and gradual restoration of neural function. At this stage, the damaged nerve axons begin to regrow in the form of growth sprouts that are referred to as “regeneration sprouts.” The mechanisms underlying the formation of these sprouts include molecular and cellular processes that promote nerve repair, such as the stimulation of axonal extension by growth factors. Specialized support cells such as Schwann cells also participate in the repair process by eliminating the residual debris at the injury site and creating myelin sheaths. Despite the potential for recovery after nerve injury, the actual degree of recovery is highly dependent on the type, site, and timing of the injury.

#### Neurotrophic factors

Since the variety of biological activities and clinical potential of neurotrophic factors (NFs) have become evident, numerous animal studies have been performed to assess various types of NFs in the treatment of nerve injuries (Terenghi, 1999). Nerve growth factor (NGF) is the principal and irreplaceable factor in the regenerative process of peripheral nerves. This creates a regenerative microenvironment for axon expansion and germination, thus providing a strong basis for nerve autorepair and regeneration. In addition, it is also important in the mechanism of recovery of the central nervous system; in particular, it assists in both the repair and proper reconnection of neurons (Zhou et al., 2023b). In addition, NGF promotes the survival and growth of sensory neurons (McMahon et al., 1995; Kaplan and Miller, 2000). Brain-derived neurotrophic factor (BDNF) is primarily distributed in the central nervous system. It can reduce neuronal apoptosis, promote the orderly formation of synapses, and support the stable growth of axons. By facilitating nerve extension, BDNF helps prevent apoptosis in a regulated manner and facilitates the formation of chemokines (Kurozumi et al., 2004; Schäbitz et al., 2007; Li et al., 2020). Glial cell line-derived neurotrophic factor (GDNF) can stimulate the proliferation of Schwann cells, increase the antioxidant capacity of neurons, and promote the survival and growth of both sensory and motor neurons (Yu et al., 1998; Airaksinen and Saarma, 2002; Höke et al., 2003). Transforming growth factor-β is involved in phenotypic changes, immunomodulation, intrinsic growth activation, and blood‒brain barrier (BBB) regulation at the site of injury in Schwann cells (Cheng et al., 2020; Ye et al., 2022). In addition, other trophic factors, such as neurotrophin-3, insulin-like growth factor 1, basic fibroblast growth factor, and vascular endothelial growth factor, also play significant roles in promoting nerve regeneration. Moreover, these factors hold potential as therapeutic agents in biomaterial-based drug delivery systems (Wan et al., 2024).

#### Role of Schwann cells in nerve repair

One of the key features of Schwann cells in nerve regeneration is their ability to dedifferentiate. They discard their myelin and transform into a more primitive, proliferative state. This change allows them to form tubular structures known as “Bungner bands,” which act as scaffolds that guide the growth of regenerating axons (Jessen and Mirsky, 2008; Liao et al., 2024). It is a type of glial cell that is typically encircled by nerve fibers to develop myelin sheaths that increase impulse conduction and safeguard axons from injury (Negro et al., 2022). These structures are essential for regenerating axons to travel their appropriate paths so that they can reconnect and restore function.

In addition to their structural roles, Schwann cells also offer guidance for axonal regeneration and the release of NGFs, among other neurotrophic factors. These elements provide critical assistance to damaged nerves, promoting axonal extension and neuronal regrowth at the site of injury. In addition, Schwann cells secrete numerous chemokines and cytokines involved in macrophage recruitment to the site of injury. Macrophages are essential for clearing cell debris and dead tissue, a process known as phagocytosis, which is crucial for preventing the formation of scar tissue that could hinder nerve repair. This removal of debris also prevents the inhibition of regenerative processes, allowing for more effective healing of the nerve (Fissel and Farah, 2021).

#### Axonal growth

During the repair process, Schwann cells assist in directional axonal growth. When injured, these cells undergo extreme morphological changes. They extend and create tube-like structures with lumens that allow regenerated axons to pass through an environment that supports regeneration. These tubes not only can act as physical conduits but can alsocan eliminate growth factors that promote axonal regeneration (Carvalho et al., 2019; Nawrotek et al., 2022).

With Schwann cells releasing growth factors, the molecules are released around the regenerating axons, whereby the axons can have the signals they need to grow in an appropriate direction. They coordinate the regenerative process by promoting growth orientation, axonal survival, and accurate reinnervation of target tissues. The sciatic nerve, like all peripheral nerves, has a critical role in repairing itself after nerve cell injury, which has led to its rapture, which is contingent upon Schwann cells helping create a scaffold as well as secreting growth-promoting factors to enable axons to regenerate and re-establish functional connections with other nerve cells. As the axon grows, the Schwann cells continue to support the axon until the regeneration process is complete. This entire process underscores the complex and essential role that Schwann cells play in nerve repair and regeneration.

#### Inflammation response

After nerve injury, cytokines and chemokines play essential roles in immune response regulation and nerve regeneration (Xing et al., 2024). Chemokines form concentration gradients that attract immune cells to the injury site, facilitating debris clearance and repair. Cytokines influence immune cell activation, proliferation, and differentiation, shaping the local microenvironment for regeneration. For example, IL-10 suppresses proinflammatory cytokines while promoting axon regeneration and myelin formation (Fregnan et al., 2012; Cheng et al., 2020). However, while acute inflammation aids in repair, chronic inflammation disrupts nerve regeneration by overexpressing proinflammatory cytokines (e.g., TNF-α and IL-1β), reducing NGF synthesis, and triggering excessive glial cell proliferation (David and Kroner, 2011). This leads to glial scar formation, which creates physical and biochemical barriers to axonal growth (He et al., 2022c). Additionally, chronic inflammation can dysregulate the immune system, causing further nerve damage. Managing inflammation effectively is crucial for optimizing nerve recovery and repair.

### Challenges in nerve regeneration

Peripheral nerve injury has been the focus of much of the research on nerve injury and its regenerative mechanisms. Although the PNS shows some spontaneous recovery after damage, the regenerative potential of nerve tissue in the PNS is quite limited. Furthermore, the growth and repair of nerve fibers require an extremely demanding microenvironment, which substantially limits the recovery process.

Autografting through surgery is the primary treatment modality for nerve injuries. This is especially true for proximal nerve injuries and long nerve defects (Lam and Leung, 2024). However, autografts have drawbacks such as donor-site morbidity, which is characterized by undesirable conditions such as infection, pain, and dysfunction at the donor site, as well limited sources that may not adequately meet the demand in terms of quantity (Lam and Leung, 2024). Furthermore, pharmacologic interventions and biologic therapies also show a number of challenges. These challenges include but are not limited to, limited efficacy in promoting long-distance axonal regeneration, the complex modulation of neural tone, and difficulties in achieving targeted delivery or sustained therapeutic effects.

These limitations in the existing treatment modalities have led to the need for novel, more efficient and safe strategies for the treatment of nerve-disruption injuries. Tissue engineering–based strategies have been shown to facilitate restoration of neural function (Sindrup and Jensen, 1999). Three-dimensional (3D) printing has emerged as a more promising solution (Liao et al., 2025). The application of 3D printing in neural repair can allow for the design of structures that are more structurally and functionally intact and precise, providing a good microenvironment for neuronal regeneration (Funakoshi et al., 1993).

## Classification and Characterization of Biomaterials Used for Preparation of Drug-Delivery Systems

Drug-delivery systems represent a technology capable of significantly enhancing the pharmacokinetic and pharmacodynamic properties of pharmaceuticals. Biomaterials play a critical role in drug-delivery systems, with their characteristics directly influencing drug release, targeting, bioavailability, and other aspects. The selection of suitable biomaterials can not only augment the therapeutic efficacy of drugs but also minimize side effects, while enabling controlled and sustained drug release.

**Figure 2:**
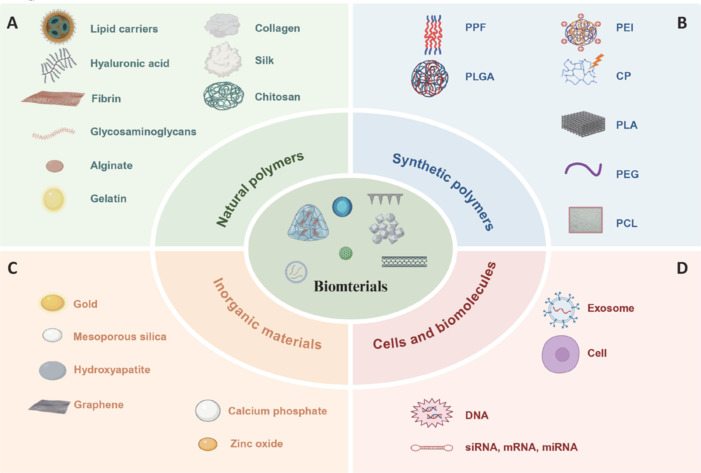
Comprehensive classification of biomaterials. (A) Natural polymers. (B) Synthetic polymers. (C) Inorganic materials. (D) Cells and biomolecules. Created with BioRender.com. CP: Conductive polymer; miRNA: microRNA; mRNA: messenger RNA; PCL: polycaprolactone; PEG: polyethylene glycol; PEI: polyethyleneimine; PLA: polylactic acid; PLGA: poly(lactic-co-glycolic acid); PPF: poly(propylene fumarate); siRNA: small interfering RNA.

These materials possess unique advantages and properties, displaying diverse behaviors in terms of biocompatibility and biodegradability (**Table 1** and **Figure 2**). Natural materials are known for their excellent biocompatibility, but their performance can be influenced by factors such as source and extraction methods. Synthetic polymers, on the other hand, can be precisely engineered through chemical synthesis to meet specific drug-delivery requirements, although their use may be associated with immune reactions. Inorganic minerals, which are characterized by distinct physical and chemical properties, offer particular advantages in specialized areas like bone tissue engineering, although their long-term safety remains a subject of ongoing research. Thus, when selecting biomaterials for drug-delivery systems, a comprehensive evaluation of the materials’ various properties is essential to ensure both safety and efficacy. With the ongoing advancements in materials science and medical technology, new biomaterials are expected to emerge, offering expanding possibilities for innovations in drug-delivery systems.

**Table 1:** Biocompatibility and degradability of different biomaterials

Biomaterial category	Biomaterial name	Biocompatibility	Biodegradability
Natural materials	Collagen	Low immunogenicity (Zheng et al., 2023); Provide support for cells (Wang et al., 2023b); Can guide tissue regeneration (Kumar et al., 2023a; Wang et al., 2023b; Zheng et al., 2023; Sowbhagya et al., 2024)	Enzymatically degradable (Wang et al., 2023b; Zheng et al., 2023; Sowbhagya et al., 2024)
	Chitosan	Promote cell adhesion, proliferation and differentiation (Kankariya and Chatterjee, 2023; Kumar et al., 2023b; Lv et al., 2023; Tang et al., 2023; Zhang et al., 2024d); Integrate well with tissues (Zhang et al., 2024d); Low toxicity (Lv et al., 2023)	Lysozyme degradable (Kankariya and Chatterjee, 2023; Kumar et al., 2023b; Lv et al., 2023; Tang et al., 2023)
	Alginate	Provide support for cells (Farshidfar et al., 2023; Tan et al., 2023); Can guide tissue regeneration cell (Mutch et al., 2024)	Alginate lyase degradable (Tan et al., 2023)
	Hyaluronic acid	Components of the extracellular matrix (Iaconisi et al., 2023; Sekar et al., 2023); Low immunogenicity (Iaconisi et al., 2023; Kotla et al., 2023)	Hyaluronidase degradable (Walvekar et al., 2024)
	Gelatin	Low immunogenicity (Mushtaq et al., 2022; Heidarian and Kouzani, 2023); Low toxicity (Mikhailov, 2023); Promote tissue repair (Zhai et al., 2023)	Protease degradable (Mushtaq et al., 2022; Mikhailov, 2023; Zhai et al., 2023)
	Silk	Facilitate tissue integration (Li and Sun, 2022; Mazurek et al., 2022); Good cell adhesion (Sultan et al., 2022; Zhou et al., 2022)	Slowly biodegradable (Mazurek et al., 2022; Hu et al., 2023a)
	Fibrin	Good affinity with various tissues (Risman et al., 2025); Blood clots forming (Gauer et al., 2023; Nencini et al., 2024; Risman et al., 2025)	Plasmin degradable (Risman et al., 2025)
	Glycosaminoglycans	Low immunogenicity (Kumar et al., 2023a; Ricard-Blum et al., 2024); Good affinity with various tissues (Khan et al., 2024a)	Enzymatically degradable (Khan et al., 2024a)
	Lipid carriers	High tissue compatibility (Go and Leal, 2021)	High biodegradability (Graván et al., 2023)
	Cells	Low immunogenicity (Chen et al., 2023c; Choi et al., 2023); Participate in tissue repair and regeneration (Wu et al., 2022a)	Metabolic process (Wang et al., 2022b), not typical degradation
	Exosomes	High tissue compatibility (Ahn et al., 2022; Kimiz-Gebologlu and Oncel, 2022; Li et al., 2024b); Low immunogenicity (Kimiz-Gebologlu and Oncel, 2022; Li et al., 2024b)	Cell uptake or macrophage cleared (Kimiz-Gebologlu and Oncel, 2022)
Synthetic polymers	PLA	Nontoxic (Ranakoti et al., 2023); Low immunogenicity (He et al., 2022b); Metabolite-friendly (Wang et al., 2022a; Ranakoti et al., 2023)	Hydrolytically degradable (Chen et al., 2023a; Ranakoti et al., 2023)
	PLGA	Low immunogenicity (Hoseinzadeh et al., 2023); Provide an attachment surface for cells (Hoseinzadeh et al., 2023; Kumar et al., 2023a)	Hydrolytically degradable, rate controllable (Hoseinzadeh et al., 2023)
	PEG	Low immunogenicity (Ibrahim et al., 2022); Low cytotoxicity (Verma et al., 2024); Good integration with tissues (Ibrahim et al., 2022; Wu et al., 2022b)	Slowly degradable under specific conditions (Ibrahim et al., 2022; Wang et al., 2023c)
	PPF	Good tissue integration (Pandele et al., 2023); Low cytotoxicity (Chen et al., 2024a; Qayoom et al., 2024)	Biodegradable (Pandele et al., 2023; Chen et al., 2024a)
	PCL	Low cytotoxicity (Backes et al., 2022; Antunovic et al., 2023); Good tissue integration (Junka et al., 2022; Antunovic et al., 2023)	Biodegradable metabolites are nontoxic (Backes et al., 2022)
	PEI	Remarkable biocompatibility (Fattahi et al., 2024)	Not well-degraded in organisms (Zhao and Zhou, 2022)
	CPs	Support the growth of a large variety of cell types (Balint et al., 2014)	Nonbiodegradability (Kiran Raj et al., 2023)
Inorganic minerals	Hydroxyapatite	Low immunogenicity (Kavitha Sri et al., 2023); High water-retention and lubricity (Kavitha Sri et al., 2023; Youseflee et al., 2023)	Biodegradable (Zhang et al., 2022b, 2024b)
	MS	Low immunogenicity (Vyas et al., 2023); Drug carrier (Thirupathi et al., 2023)	Stable chemical properties and poor degradability (Thirupathi et al., 2023)
	Gold	Inert (Yañez-Aulestia et al., 2022); Low immunogenicity (Yang et al., 2022d); Good tissue compatibility (Yañez-Aulestia et al., 2022; Dykman et al., 2025)	Nonbiodegradable (Mutalik et al., 2023; Dykman et al., 2025)
	CaP	Good osteoconductivity and cytocompatibility (Chen et al., 2023f; Lukina et al., 2023; Pylostomou et al., 2023)	Slowly degradable in body fluids (Qiu et al., 2022; Chen et al., 2023f)
	Zinc oxide	Low immunogenicity (Asif et al., 2023); Low toxicity (Asif et al., 2023); Antibacterial (El-Saadony et al., 2024; Okaiyeto et al., 2024)	Biodegradable (Tang et al., 2024a)
	Graphene	Good biocompatibility (Liao et al., 2018)	Considerable biodegradability (Chen et al., 2017)

CaP: Calcium phosphate; CP: conductive polymer; MS: mesoporous silica; PCL: polycaprolactone; PEG: polyethylene glycol; PEI: polyethyleneimine; PLA: polylactic acid; PLGA: poly(lactic-co-glycolic acid); PPF: poly(propylene fumarate).

### Classification and biological properties of biomaterials

#### Natural materials

Collagen is the most abundant protein in animals. It shows excellent biocompatibility with human tissues, causing no obvious immune rejection and thus creating a stable environment for drug delivery (Sarrigiannidis et al., 2021). Collagen can be degraded by enzymes in the body into amino acids that can participate in metabolism, leaving no residues or side effects (Wosicka-Frąckowiak et al., 2024). The unique triple-helix structure of collagen enables it to bind to drugs in various ways. Moreover, by modification, collagen can be linked to targeting ligands to achieve targeted delivery. Simultaneously, collagen can provide a favorable microenvironment for cells, promoting tissue repair and regeneration (Kusnadi et al., 2024). Additionally, the slow-release properties of collagen allow regulation of the drug-release rate, prolonging the duration of drug action and reducing the frequency of administration (Manuel et al., 2021). In a drug-delivery system, collagen can be employed in various dosage forms such as microspheres and nanoparticles. For example, encapsulating drugs in collagen microspheres can enable the slow release of drugs. Due to the structural similarity of collagen to human tissues, cells can adhere, proliferate, and differentiate on the collagen scaffold, which is highly beneficial for tissue engineering and local drug delivery (Rana et al., 2023).

Chitosan is a natural cationic polysaccharide obtained by deacetylation of chitin (CaterinaValentino et al., 2025). It can be used in drug-delivery systems. As a natural polysaccharide, chitosan shows excellent biocompatibility with human tissues and cells (Rangel-Garcia et al., 2025), causing no obvious immune response or toxicity (Iacob et al., 2021). It can be degraded by enzymes in the body into oligosaccharides that are absorbed or excreted (Baharlouei and Rahman, 2022). Chitosan’s good water solubility, pH responsiveness, and other physicochemical properties can facilitate precise drug release according to the environment and the formation of stable complexes with drugs (Nazemoroaia et al., 2025). Moreover, modifications of chitosan can allow it to be linked to targeting ligands to achieve targeted delivery (Niculescu and Grumezescu, 2022). Through its mucoadhesive properties, chitosan can also promote drug absorption (Loo et al., 2022). Moreover, chitosan can be employed in various dosage forms to regulate the drug-release rate, achieve sustained and controlled release, reduce the dosing frequency, and improve patients’ medication compliance (Jeencham et al., 2019; Patel et al., 2021).

Alginate is a natural polysaccharide-based biopolymer material extracted from brown algae and other algae (Adamiak and Sionkowska, 2023). Alginate can be used for drug-delivery systems due to its multiple characteristics (Zhang et al., 2024e). It shows good biocompatibility (Xie et al., 2022), coexists harmoniously with human tissues, and does not trigger severe immune and inflammatory reactions (Liu et al., 2022). It can also be slowly degraded by enzymes in the organism, and the degradation products can participate in metabolism and be eliminated (Farshidfar et al., 2023). Alginate has controllable gel properties. In the presence of divalent cations, it can rapidly form a gel to encapsulate drugs, and the drug-release rate can be precisely regulated by adjusting the relevant conditions (Hurtado et al., 2022; Tomić et al., 2023; Wathoni et al., 2024). Alginate is also easy to modify and functionalize. The active groups on the molecular chain can be linked to targeting groups to achieve targeted drug delivery, or linked to special functional groups to endow responsiveness (He et al., 2022a). In addition, alginate shows low toxicity and is a low-cost material that is highly safe and conducive to large-scale production, reducing the cost of medication for patients (Rana et al., 2024).

Hyaluronic acid is a linear polysaccharide widely present in the connective tissues of the human body (Graciela et al., 2023). It has good biocompatibility and high hydrophilicity and can form hydrogels in the body. The unique feature of hyaluronic acid is its ability to bind to receptors on the cell surface, conferring certain cell-targeting properties (Lis et al., 2023). In drug-delivery systems, hyaluronic acid can be used as a drug carrier to deliver drugs to specific cells or tissues (Sallum et al., 2024). For example, binding anti-cancer drugs to hyaluronic acid can facilitate the entry of the drugs into tumor cells and improve the therapeutic effects of the drugs. Hyaluronic acid can be degraded by hyaluronidase in the body, and its degradation rate can be controlled by adjusting its molecular weight and chemical modification (Kuang et al., 2021).

Gelatin is a partial hydrolysis product of collagen that retains some of the excellent properties of collagen, such as biocompatibility and degradability (Nii, 2021). The advantages of gelatin lie in its good water solubility and plasticity. Its gelation properties can be adjusted by changing conditions such as temperature and pH (Mujawar et al., 2025). For drug delivery, gelatin can be used to prepare gels (Maghsoudi et al., 2025), and microcapsules. For example, microcapsules with gelatin as the wall material can effectively protect drugs from the external environment and slowly release drugs into the body through enzymatic hydrolysis (Ugrinovic et al., 2024).

Silk, especially silk proteins such as silk fibroin, is an ideal material for drug-delivery systems in nerve regeneration due to its excellent biocompatibility, biodegradability, and mechanical properties (Chen et al., 2023b). The porous structure and high surface area of silk enable it to effectively load drugs and control their release, thus promoting nerve repair (Fernández-González et al., 2024). Silk can be used to fabricate nerve conduits, nanoparticles, and hydrogels, providing physical support for nerve regeneration and enabling sustained drug release (Hassan et al., 2024; Quan et al., 2025). Silk materials have been shown to allow the proliferation of nerve cells as well as recovery of their functions *in vitro* and in animal experiments (Yang et al., 2022a). These materials have also been shown to be safe and effective in preliminary clinical trials (Siritientong et al., 2014; Fine et al., 2015). Although the degradation rate of these materials needs to be optimized and their drug-release regulation and long-term safety require further investigation, silk is a promising biomaterial for nerve regeneration (Wani et al., 2022; Zhou et al., 2022).

Being a natural biomaterial, fibrin is ideal for drug-delivery systems for nerve regeneration, since it can promote cell adhesion and shows excellent biocompatibility and biodegradability (Sanz-Horta et al., 2023; Li et al., 2024c). The fibrous network structure of fibrin can also mimic the extracellular matrix and provide support for nerve cells (Rojas-Murillo et al., 2022). Moreover, controlled release of growth factors (such as nerve growth factor [NGF], brain-derived neurotrophic factor [BDNF]) or drugs loaded onto fibrin can promote the growth of axons and repair nerves (Pereira et al., 2023). Fibrin hydrogels and scaffolds have shown good repair effects in models of peripheral nerve injury and spinal cord injury, significantly promoting nerve-function recovery (Bozorgi et al., 2024). Despite the need for optimization of mechanical properties and degradation rate, fibrin has shown a wide range of applications in nerve regeneration (Shi et al., 2022; Narayanaswamy et al., 2023).

GAGs have been suggested to be ideal biomaterials for nerve-regeneration drug-delivery systems due to their very good biocompatibility, biodegradability, and similarity to their extracellular matrix (Chen et al., 2023g; Waseem et al., 2023). GAGs can promote neural repair by modulating cell-signaling pathways, limiting glial-scar development, and enhancing axon regeneration (Deng et al., 2023). Moreover, GAGs can undergo chemical modifications or be incorporated with other biomaterials (e.g., collagen, fibrin) to form multifunctional scaffolds or hydrogels for sustained release of growth factors (e.g., NGF, BDNF) or drugs (Wieboldt and Läubli, 2022; Segars and Trinkaus-Randall, 2023). GAG-based materials have been shown to exhibit substantial nerve-regeneration and functional-recovery effects in spinal cord and peripheral nerve-injury models, demonstrating broad application prospects (Siddiqui et al., 2022; Yang et al., 2024b).

Lipid carriers, which are composed of phospholipids, cholesterol, and other lipid molecules, also exhibit exceptional biocompatibility. In addition to their ability to encapsulate and deliver both hydrophilic and lipophilic drugs, enhancing the stability of these drugs and prolonging their circulation time *in vivo*, these carriers can also fuse with cell membranes or enter cells via endocytosis, thereby significantly improving drug bioavailability. Consequently, lipid carriers offer distinct advantages in the delivery of small-molecule drugs, protein therapeutics, and gene therapies (Dymek and Sikora, 2022). Liposomes and lipid nanoparticles (LNPs) are the most commonly used lipid carriers. In comparison with liposomes, LNPs can incorporate ionizable lipids, enabling them to bind to DNA or RNA under specific pH conditions, which represents an irreplaceable advantage in gene drug delivery (Eygeris et al., 2022).

From the perspective of biocompatibility, the use of cells such as red blood cells (Wang et al., 2022b; Chen et al., 2023c), mononuclear macrophages, tumor cells (Choi et al., 2023), and bacterial cells as drug carriers can greatly improve the biocompatibility of drugs (Wang, 2023). Cells are part of an organism, and they have good adaptability to the organism, which can reduce the possibility of immune rejection of drugs by the body (Xu et al., 2022b; Fang et al., 2024). For example, the use of red blood cells as a drug-delivery system has been widely studied due to the remarkable properties of these cells, including their inherent biocompatibility, low immunogenicity, flexibility, and long systemic circulation (Han et al., 2018). Cell-based drug-delivery systems also show certain advantages in terms of degradability (Xiao et al., 2022). Some cell–based drug-delivery systems can degrade in specific environments to release drugs (Rong et al., 2022). For example, waterborne polyurethane nanoparticles prepared from isophorone diisocyanate and other materials can rapidly swell and break down to release drugs in the intracellular reductive-acidic simulated environment (Nguyen et al., 2023).

Exosomes have excellent biocompatibility (Sadeghi et al., 2023). Exosomes are nanovesicles secreted by cells; they carry substances such as nucleic acids and proteins and play roles in cell communication and material transfer in the body (Zhang et al., 2022a; Erana-Perez et al., 2024). Their low immunogenicity and low toxicity reduce the likelihood of immune rejection, potentially ensuring better compatibility with the organism (Ferreira et al., 2022). Moreover, exosomes can serve as an excellent new-type nanoscale carrier for carrying protein-based drugs and achieving targeted drug delivery (Rajput et al., 2022). In terms of degradability, exosomes are biodegradable; thus, they can be gradually degraded by the organism after completing the drug-delivery task and will not accumulate in the body to cause long-term adverse effects (Tenchov et al., 2022). For example, in cancer treatment, exosomes have been used as natural nanomaterials with many functional proteins on their membrane surface and substances such as RNA and DNA in their cavity (Lu et al., 2024). Due to their high biocompatibility, excellent penetration ability, and ease of modification, exosomes have shown outstanding potential in delivering therapeutic molecules for cancer diagnosis and treatment (Lu et al., 2024). Exosomes can also be used in bio-derived drug-transportation and protection devices for the treatment of diseases such as bacteria- and virus-related diseases and cancer (Zhang et al., 2022f).

#### Synthetic polymers

*Polylactic acid*

PLA is a biodegradable polyester polymerized from lactic acid monomers. PLA shows good biocompatibility, mechanical properties, and processability (Sánchez-Cepeda et al., 2024). Its biodegradation mainly occurs through the hydrolysis of ester bonds. In the body, it can be gradually degraded into lactic acid, which is then metabolized by the human body through the tricarboxylic acid cycle (Bakhtiari et al., 2025). PLA can be employed in various dosage forms such as microspheres and nanofibers for drug delivery (Cárdenas-Aguazaco et al., 2023). For example, encapsulation of antibiotics in PLA microspheres can facilitate long-acting slow release of antibiotics, reducing the frequency of administration and improving patient compliance (Le et al., 2024).

*Poly(lactic-co-glycolic acid)*

PLGA, a copolymer of lactic acid and glycolic acid, combines the advantages of PLA and polyglycolic acid. The biodegradation rate of PLGA is faster than that of PLA (Ciocîlteu et al., 2023), and the degradation and drug-release rates of PLGA-based systems can be controlled by adjusting the ratio of lactic acid to glycolic acid (Qi and Liu, 2012). PLGA is one of the most widely used synthetic polymers in drug-delivery systems (Naskar et al., 2021). For example, encapsulation of anti-tumor drugs in PLGA nanoparticles can achieve targeted delivery and controlled release of drugs, increasing the concentration of drugs in tumor tissues and reducing the toxicity of drugs to normal tissues.

*Polyethylene glycol*

PEG is a hydrophilic synthetic polymer with good biocompatibility and water solubility (Huang et al., 2024). PEG can be covalently bonded to drug molecules or other carrier materials, prolonging the circulation time of drugs, improving drug stability, and reducing immunogenicity (Gökçe Kocabay and and İsmail, 2021). For example, modifying the surface of liposomes with PEG can reduce liposome uptake by the reticuloendothelial system, prolong the circulation time of liposomes in the blood, and thereby increase the possibility of drugs reaching the target tissue (Zhou et al., 2023a; Liu et al., 2024a). PEG is essentially not degraded in the body and is mainly excreted from the body through the kidneys (D’Souza A and Shegokar, 2016).

*Poly(propylene fumarate)*

PPF is a biodegradable synthetic polymer that has emerged as an ideal biomaterial for nerve-regeneration drug-delivery systems due to its excellent mechanical properties, processability, and biocompatibility (Pandele et al., 2023; Chen et al., 2024a). PPF can be processed into nerve conduits or 3D-printed scaffolds through thermal or photo-curing techniques, providing physical support for nerve regeneration (Kirillova et al., 2022; Qayoom et al., 2024). Loading of NFs or anti-inflammatory drugs onto PPF allows controlled release of these therapeutic agents, promoting axonal growth and functional recovery (Periferakis et al., 2024). PPF-based materials have shown good biocompatibility and repair effects in peripheral nerve injury and spinal cord injury models (Liu et al., 2025). Although further optimization of the degradation rate and surface functionality of PPF is needed, PPF holds broad application potential in the field of nerve regeneration (Xu et al., 2024).

*Polycaprolactone*

PCL is another biodegradable synthetic polymer that has become an ideal biomaterial for nerve-regeneration drug-delivery systems due to its excellent mechanical properties, controllable degradation rate, and good biocompatibility (Antunovic et al., 2023; Raza et al., 2024). PCL can be processed into nerve conduits, nanofiber scaffolds, or microspheres using techniques such as electrospinning and 3D printing, providing structural support for nerve regeneration (Murab et al., 2023; Yan et al., 2024). Loading of NFs (e.g., NGF, BDNF) or drugs onto PCL can enable sustained release of these agents, promoting axonal growth and functional recovery (Pontinha et al., 2023). PCL-based materials exhibit good biocompatibility and regenerative effects in the repair of peripheral nerve injuries and spinal cord injuries (Rahimkhoei et al., 2023; Tang et al., 2024b). Although the hydrophobicity of PCL may limit cell adhesion (Li et al., 2023b), surface modifications (e.g., grafting bioactive molecules) can significantly enhance its performance, conferring it with broad application prospects in the field of nerve regeneration (Haider et al., 2023; Ibrahim et al., 2023).

*Polyethyleneimine*

PEI is a cationic polymer with exceptional gene-delivery capabilities. PEI can efficiently bind to DNA or RNA through electrostatic interactions, forming stable nanoparticles that enhance the stability of nucleic acid therapeutics and protect them from nuclease degradation. Moreover, PEI can also facilitate cellular uptake of these therapeutic agents and enable efficient endosomal escape via the proton-sponge effect, thereby significantly improving the bioavailability of gene therapeutics (Akinc et al., 2005; Nel et al., 2009; Virgen-Ortíz et al., 2017). Consequently, PEI has demonstrated unique advantages in gene therapy, RNA interference, and vaccine delivery. Among the various PEI carriers available at present, linear PEI and branched PEI are the most commonly used. In comparison with linear PEI, branched PEI shows superior buffering capacity, allowing more effective nucleic acid release. However, its relatively higher cytotoxicity necessitates structural modifications to enhance its safety and delivery efficiency in practical applications (Fattahi et al., 2024).

*Conductive polymers*

CPs are composed of conjugated molecular chains, which offer excellent electrical conductivity and biocompatibility (Yi et al., 2023). Common conductive polymers include poly(3,4-ethylenedioxythiophene), poly(3-hexylthiophene), polypyrrole, and polyaniline (Xuan et al., 2023). These materials can facilitate electrical signal transmission to promote nerve regeneration and also serve as carriers for both hydrophilic and hydrophobic drugs, enhancing drug stability and enabling controlled release (Collazos-Castro et al., 2016). Consequently, CPs have shown unique advantages in delivering small-molecule drugs, protein therapeutics, and gene therapy agents. In the future, optimizing the structure and functionality of these materials can further enhance the applications of CPs in precision medicine and personalized therapy, providing more efficient and safer drug-delivery systems for modern medicine.

#### Inorganic minerals

*Hydroxyapatite*

Hydroxyapatite is an inorganic mineral with good biocompatibility (Verma et al., 2023). Its chemical composition is similar to that of the mineral components of human bones and teeth (Niziołek et al., 2023). Hydroxyapatite has high bioactivity and can form chemical bonds with surrounding tissues (Ambrosio et al., 2023; Qian et al., 2023). In drug delivery systems, hydroxyapatite can be used as a drug carrier, especially for bone tissue engineering and local drug delivery (Hu et al., 2022; Mashak et al., 2022). For example, loading growth factors or antibiotics on hydroxyapatite scaffolds can promote bone tissue repair and prevent infection (Chen et al., 2023e; Xie et al., 2023). In addition, hydroxyapatite can promote bone repair without causing significant immune rejection (Soriente et al., 2022; Fendi et al., 2024). The degradation rate of HA in the body is relatively slow, mainly through cell-mediated dissolution and chemical dissolution (Karacan et al., 2021).

*Mesoporous silica*

MS has a large specific surface area, a regular pore structure (Sarkar et al., 2023), and good chemical stability (Skwira et al., 2023). It has good biocompatibility and can efficiently load drugs in drug delivery systems (Budiman et al., 2024; Lin et al., 2024; Sha’at et al., 2024). The pore size and shape of MSs can be controlled by adjusting the synthesis conditions, enabling the loading of drug molecules of different sizes (Yang et al., 2023b; Kaur et al., 2024). For example, loading the anticancer drug doxorubicin in MS nanoparticles can improve the drug loading capacity and release controllability (Chang et al., 2022; Skwira et al., 2023). The degradation rate of MS in the body is slow, but its clearance rate in the body can be adjusted through surface modification (Djayanti et al., 2023; Li et al., 2023a; Zhu et al., 2023b; Heidari et al., 2024). For example, introducing biodegradable chemical bonds or groups on the surface of MS can accelerate its degradation and clearance in the body (Skwira et al., 2023; Yang et al., 2023a).

*Gold*

Owing to its excellent biocompatibility, chemical stability, and unique optical properties, gold has emerged as an innovative biomaterial for nerve regeneration drug delivery systems (Lin et al., 2022; Sakthi Devi et al., 2022). Gold nanoparticles can be surface modified to load drugs or NFs (e.g., NGF and BDNF) and utilize their photothermal effects to achieve precise controlled release, promoting neural repair (Jung and Nam, 2022). Additionally, gold nanowires or gold-based composite materials can be used to fabricate neural interfaces or conductive scaffolds, supporting electrical signal transmission and axonal regeneration (Dykman et al., 2025). Studies have shown that gold-based materials exhibit good biocompatibility and functional recovery effects in the repair of peripheral nerve injuries and spinal cord injuries (Martínez-Cuazitl et al., 2023; Silveira et al., 2023). Although further research is needed to assess their long-term biosafety, gold-based materials hold significant potential in the field of nerve regeneration (Yañez-Aulestia et al., 2022).

*Calcium phosphate*

CaP could be an ideal candidate for drug delivery systems in nerve regeneration because of its excellent biocompatibility, biodegradability, and osteoconductive properties (Chen et al., 2023f). Owing to its chemical resemblance to the mineral component of bone and teeth, it is an appropriate candidate for biomedical applications (Zhang et al., 2022e). CaP-based materials can be formulated into nanoparticles, scaffolds, or coatings for the localized delivery of growth factors, neurotrophic factors, or small-molecule drugs for nerve regeneration at the injury site (Qiu et al., 2022; Uskoković, 2025). These systems allow delivery of drugs in a sustained and controlled manner, thereby enhancing neuroregeneration while reducing off-target effects (Pylostomou et al., 2023). Furthermore, the versatile degradation rate of CaP in combination with its ability to bind and stabilize biomolecules offers the potential to finely tune drug release kinetics (Lukina et al., 2023; Fan et al., 2024). In addition, the surface of CaP biomaterials can also be functionalized with peptides or other bioactive molecules to improve cell adhesion, proliferation and differentiation while assisting in tissue repair and regeneration in damaged neural environments (Kouhi et al., 2024). Overall, CaP-based drug delivery systems hold significant potential for advancing nerve regeneration therapies.

*Zinc oxide*

Owing to its unique physicochemical characteristics, biocompatibility, and multifunctionality, ZnO has attracted considerable interest as a biomaterial for drug delivery systems in nerve recovery in recent years (Rashki et al., 2023). In particular, ZnO NPs have been reported to possess an advantageous drug-loading capacity and controlled release behavior, as well as the ability to pass through the BBB, suggesting that they enable the transport of therapeutic agents (e.g., neurotrophic factors, antioxidants, and anti-inflammatory drugs) to sites of neural injury (Costa et al., 2023; Gupta et al., 2023; Eixenberger et al., 2025). Moreover, ZnO has natural neuroprotective and antimicrobial characteristics, aiding in the reduction of oxidative damage and the prevention of infections during regeneration. They can be functionalized by targeting ligands or biomolecules due to their tunable surface chemistry, improving their specificity and therapeutic efficacy (Li et al., 2023g; Wu et al., 2024). In addition, mechanical (stress) stimulation of neural cell growth and differentiation can be promoted with the help of the piezoelectric properties of ZnO under the influence of mechanical stimulation, thus facilitating tissue repair (Younas et al., 2023). Overall, ZnO-based drug delivery systems hold great promise for advancing nerve regeneration therapies.

*Graphene*

Graphene and its chemical derivatives have been widely used in many fields, from biomedical research drug delivery to bioelectronics and tissue engineering. Graphene derivatives can be classified into two groups: chemically modified graphene (CMG) and functionalized graphene (FG). CMGs include mainly graphene oxide, reduced graphene oxide, and multilayer graphene oxide. These materials exhibit better assembly capabilities and lower costs (Yuan et al., 2018). FGs are modified mainly through covalent and noncovalent functionalization to increase their solubility, dispersibility, conductivity, and applicability in drug delivery (Sagadevan et al., 2021). At present, graphene-based materials exhibit excellent performance in bone and skin tissue engineering, leveraging their outstanding biological, thermal, and mechanical properties, as well as their biocompatibility (Ghuge et al., 2017; Dias et al., 2021). Notably, the potential of graphene oxide and reduced graphene oxide for nerve regeneration has also been demonstrated, primarily because of their excellent electrical conductivity (Huang et al., 2021).

## Different Repair Materials in Central Nervous System and Peripheral Nervous System

The main objectives of the design strategy for PNS damage-repair materials are to reconstruct the physical pathway of axon regeneration and optimize the regeneration microenvironment (Yao et al., 2025). As mentioned earlier, Schwann cells rapidly dedifferentiate and secrete NFs to form a “regeneration conduit” after PNS injury. Therefore, the repair materials should facilitate Schwann cell migration and axonal guidance, which is usually achieved with biodegradable nerve conduits or fiber-aligned materials. These materials promote regeneration through mechanical guidance and localized NF release.

In contrast, the design strategies for injury-repair materials for the CNS differ significantly from those for the PNS, requiring adaptation to highly complex environmental challenges, particularly within the gray matter. CNS gray matter is primarily composed of neuronal cell bodies, and damage to the CNS gray matter leads to critical issues such as neuronal death, synaptic loss, and uncontrolled local inflammation. At present, no gold standard approaches are available for treating damaged neurons, and conventional medicine only provides protective strategies to alleviate the symptoms of chronic injury (Zheng and Tuszynski, 2023). Moreover, conventional materials struggle to penetrate the dense neural cell network and physiological barriers in the gray matter, often resulting in glial scarring (He et al., 2020b). Therefore, therapeutic approaches using biomaterials for gray matter repair should focus on neuroprotection, synaptic reconstruction, scar inhibition, and anti-inflammatory regulation.

## Functional Strategies Using Biomaterials

**Figure 3:**
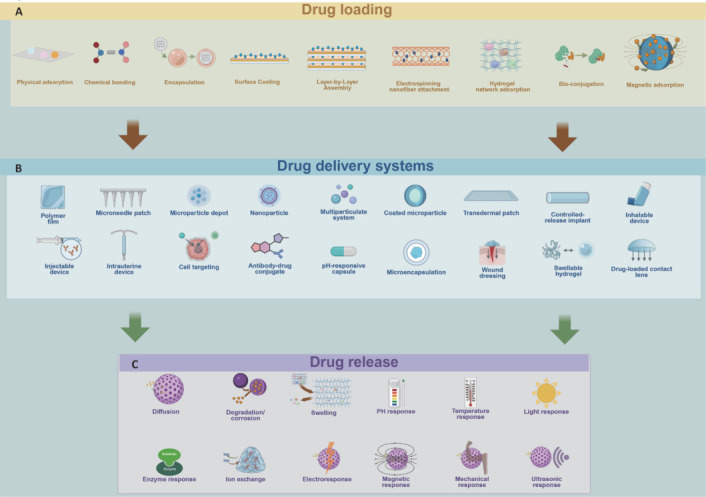
Overview of drug loading, delivery systems, and release mechanisms. (A) Different drug loading methods. (B) Different drug delivery systems. (C) Different drug release mechanisms. Created with BioRender.com.

The intelligent design of biomaterials has endowed drug-delivery systems with precise spatiotemporal control, with the core functional strategies advancing across three progressive dimensions: (1) high-efficiency drug-carrier compatibility and loading; (2) intelligent navigation across biological barriers; and (3) microenvironment-responsive programmed release. The synergistic optimization of these strategies can significantly enhance therapeutic efficacy while reducing systemic toxicity and improving patient compliance (**Figure 3**).

### Drug-loading strategies

Drug loading, which involves the attachment of the drug to the biomaterial, is the first step in the development of a drug-delivery system. The factors affecting drug loading mainly include the different biological materials and loading techniques.

Different biomaterials present distinct benefits in drug loading. For example, polymers such as PLGA are commonly utilized because of their compatibility with biological systems, their ability to degrade safely within the body, and their capacity to form nanoparticles that can encapsulate a variety of hydrophilic and hydrophobic drugs (Pinto et al., 2022; Su et al., 2022). In contrast, hydrogels are particularly valued for their high water content and swelling properties, which make them ideal for loading larger molecules such as proteins and peptides (Freedman et al., 2022; Sanjanwala et al., 2022). Moreover, the differences among biomaterials are also reflected in the distinct chemical modifications applied to them. Surface functionalization, as a prevalent chemical modification, enhances the biocompatibility and stability of nanocomponents (Zhang et al., 2024a). For example, materials modified with surface functionalization using PEG exhibit enhanced stability (Amin and Boateng, 2023). Additionally, chemical modifications such as the introduction of functional groups, grafting bioactive molecules, or integration of stimuli-responsive elements can further enhance drug-loading capacity and controlled release.

Various techniques, such as physical adsorption, chemical bonding, and encapsulation, have been extensively studied and applied in this context (Lilja et al., 2013; Lee et al., 2019). Some of the more recent and stable technologies, such as magnetic nanoparticle adsorption, have also been validated in a previous study (Liu et al., 2023b). In a study by Choi et al. (2018), bio-conjugation was found to yield better drug-loading capacity and stability than physical adsorption and charged adsorption. For example, nucleic acid therapeutics such as small interfering RNA (siRNA), messenger RNA (mRNA), and antisense oligonucleotides (ASOs) are prone to degradation, clearance, and immune activation *in vivo*. Chemical modifications can be used to enhance their stability and therapeutic efficacy, while specialized delivery systems can facilitate efficient and targeted transport. The key types of chemical modifications include nucleoside base modifications and phosphate backbone modifications, as outlined in this review (Gupta et al., 2021). Meanwhile, delivery vectors for nucleic acids can be broadly categorized into viral and non-viral systems, which will be discussed in detail in the following sections.

### Targeted drug-delivery strategies

The primary goal of targeted neurodrug delivery is to precisely transport therapeutic agents across the blood–spinal cord barrier and the blood–brain barrier (BBB) while minimizing damage to healthy neural tissues. The existing strategies often rely on antibodies or peptides to recognize specific neural cell receptors. For instance, Qian et al. (2024) leveraged the neurotropic properties of the rabies virus glycoprotein 29 and combined it with short-chain hyaluronic acid, which exhibits a high affinity for CD44-expressing cells, to achieve targeted delivery of curcumin. This system demonstrated a dual-phase neuroprotective effect: early inhibition of immune cell infiltration and inflammatory cascades, followed by protection against inflammation-induced apoptosis in the later stages of neural regeneration.

However, the BBB remains one of the biggest challenges in targeted CNS drug delivery. As a monolayer of tightly connected endothelial cells, the BBB restricts the entry of most exogenous compounds into the CNS while actively exporting molecules back into systemic circulation through adenosine triphosphate-binding cassette transporters (Pinto et al., 2022). Certain endothelial cell transporters allow the passage of small molecules such as amino acids, glucose, and organic anions (Eleftheriadou et al., 2020). However, leveraging these endogenous pathways for targeted drug delivery remains an area of active research. One promising approach is intranasal administration, which can effectively bypass the BBB (Duan et al., 2024; Liu et al., 2024c). Another strategy involves nanoparticle functionalization with ligands that target brain endothelial receptors. This method can enable drug transport through adsorptive-mediated, transporter-mediated, or receptor-mediated endocytosis (Rafati et al., 2024).

In comparison with traditional nanoparticle-delivery systems, cell-based and extracellular vesicle therapies that have emerged in recent years represent a more biocompatible alternative. With the growing interest in cell-based and extracellular vesicle therapies, the role of engineered exosomes in targeted therapy for neural tissue deserves consideration. Cellular biosynthesized exosomes have a membrane structure consisting of 30–100 nanomaterials, and they can be surface-modified with molecules that bind to specific receptors of BBB epithelial cells such as transferrin, low-density lipoprotein receptor family, intracellular adhesion molecules, insulin and glucose receptors, thereby efficiently crossing biological barriers and allowing precise delivery of drugs or genetic material to brain tissue (Li et al., 2023f; Rehman et al., 2023). He et al. (2020a) co-incubated macrophage-derived exosomes with the hydrophobic drug curcumin to generate engineered exosomes targeting the brain ischemia-reperfusion area. Furthermore, exosomes possess the ability to target specific cells. Therefore, utilizing their capacity to deliver nucleic acids to target cells to suppress neuroinflammation or promote nerve regeneration is a promising approach in precision medicine. Li et al. (2023d) observed that plasma-derived exosomes carrying miR-20b-3p significantly improved peripheral neuropathy in type I diabetic rats. Jiang et al. (2024a) used exosomes modified with the targeting peptide RVG29 to load miR-133b and thereby improve Parkinson disease. Unlike synthetic nanoparticles, exosomes can be derived from a patient’s own cells, significantly reducing the potential for immunogenicity and cytotoxicity. Furthermore, while exosomes possess intrinsic BBB-penetrating and targeted-delivery properties, their RNA cargo itself can serve as a therapeutic agent, further enhancing their clinical potential. However, the clinical translation of these findings faces key challenges, including scalable production, precise molecular engineering, and optimization of delivery efficiency. Moving forward, integrating synthetic biology for enhanced exosome loading, leveraging AI-driven screening for optimized targeting peptides, and developing smart-controlled release systems could propel the clinical application of exosome-based precision neurotherapeutics.

### Advancements in controlled release technology *in vivo*

After incorporation of the drug into the biomaterial, its release into the body needs to be meticulously regulated to ensure the intended therapeutic outcome. The release process is typically influenced by the interplay of the drug, the biomaterial, and the biological environment in which it is placed. Various methods, such as diffusion, material degradation, affinity-based release, and stimuli-responsive mechanisms, can be employed to achieve controlled and precise drug release (Shan and Wu, 2024). Continuous release is a common mode of biomaterial drug release; this process mainly occurs through diffusion- or erosion-controlled release, so it lacks advantages in specific biological microenvironments. The ongoing advancements in technology have led to an endless stream of new methods to control drug release, such as the use of nano-emulsions, micro-emulsions, transfer bodies, alcoholsomes, nano-emulsions, and micron mixtures (Dumitriu Buzia et al., 2023). Currently, an emerging drug-delivery approach involves the use of stimuli-responsive biomaterials, which release drugs in response to specific environmental triggers. These include drug release through various internal stimuli, including redox reactions, pH changes, and enzymes, as well as external stimuli such as temperature, ultrasound, magnetic fields, light, voltage, and mechanical responses; this approach has shown progress in the treatment of tissue regeneration (Ding et al., 2022; Zhang et al., 2022c).

## Types and Comparison of Neuroregenerative Drug-Delivery Systems

The treatment of brain, spinal cord, and peripheral nerve injuries faces dual challenges posed by the BBB and targeted delivery. Traditional therapeutic approaches often struggle to achieve efficient and precise delivery of drugs or genes, making both local and systemic delivery strategies key areas of research. The differences in delivery efficiency, safety, and targeting capability between viral and non-viral vector systems directly influence therapeutic outcomes. This section of the review will analyze the characteristics of these strategies and vectors, providing a theoretical basis for treatment optimization.

### Systemic delivery *versus* localized delivery

Local delivery and systemic delivery have unique strengths and weaknesses that require consideration in therapeutic contexts. Local delivery of drugs can facilitate administration of the therapeutic agent at the sites where the drug is required. Local delivery provides a higher concentration of the drug at/in the site of injury, typically minimizing systemic side effects (Bhunia et al., 2023). This makes it extremely suitable for treating localized conditions such as peripheral nerve damage, spinal cord injuries, or brain tumors (Vogelbaum et al., 2022; Guillemot-Legris et al., 2023; Hua et al., 2024; Qin et al., 2024). Nasal drug delivery is a promising administration route due to its ease of use and patient-compliance characteristics; using this approach, the molecule can directly target the CNS while evading the BBB. Nasal drug delivery would thus be an increasingly attractive route of administration ideally suited for clinical practice given its simplicity and significant patient compliance. It also has the ability to cross the BBB as well as deliver drugs to the brain through the olfactory and trigeminal nerves and execute therapeutic effects (Lee and Minko, 2021; Gotoh et al., 2024; Khan et al., 2024b). The existing studies have explored intranasal delivery of insulin-like growth factor to the brain and showed that this approach exhibits significant neuroregenerative effects following cerebral ischemia (Liu et al., 2001; Cao et al., 2024). However, local delivery often requires more invasive procedures—such as surgery, intracerebral injection, ventricular injection, and intraspinal injection—to deliver drugs directly to the target area and increase local drug concentration. These methods carry surgical risks and potential complications, such as infection, increasing the overall complexity and risk of treatment (Kalam et al., 2017; Fonseca-Santos et al., 2020). Intracerebral injection can deliver the drug directly to the lesion site, but due to the complex anatomy of the brain, the injection process may damage the surrounding neural tissues (Tomiyama et al., 1994; Bi et al., 2023). Ventricular injections, on the other hand, can diffuse the drug throughout the CNS through the circulation of cerebrospinal fluid, but the distribution and targeting of the drug are more difficult to control in this approach (Yu et al., 2020b; Li et al., 2024d). Intraspinal injections (e.g., intrathecal injections) can deliver drugs directly to the spinal cord for the treatment of spinal cord injuries and spinal cord disorders, but these injections require precise localization and maneuvering skills to avoid damage to the spinal cord (Chaterji et al., 2021; Gimarc et al., 2021). In addition, local treatment has a limited scope and is not suitable for treating widespread or systemic diseases.

In contrast, systemic delivery involves the administration of drugs through the bloodstream, making it suitable for treating diseases that are widely distributed. Systemic delivery is simpler to perform, typically involving non-invasive methods such as oral ingestion or intravenous injection, and it can be easily applied to various therapeutic scenarios. Although oral drug delivery is convenient, special delivery systems are needed to protect nucleic acids and promote their absorption because nucleic acids are easily degraded by nucleases in the gastrointestinal tract, and their absorption is less efficient (Yu et al., 2020a; Sung et al., 2022). Delivery systems based on nanoparticles or liposomes have shown the potential to significantly improve the stability and bioavailability of nucleic acids (Attarwala et al., 2018; Wei et al., 2023). However, even if the drug is well-protected before absorption, once it enters the bloodstream, the dilution effect can lead to insufficient drug concentration, especially when the drug needs to cross the BBB, thereby limiting its effectiveness. Additionally, systemic delivery may lead to unwanted side effects because the drug affects not only the target area but also non-target tissues.

The pursuit of more effective and safer treatments for neurodegenerative diseases and neurological disorders has led to numerous studies on advanced drug-delivery systems. These systems, which include lipid- and polymer-based nanoparticles, gel-based systems, and drug conjugates, are highly biocompatible (Rani et al., 2023; Xu et al., 2023c; Yin et al., 2024; Zhang et al., 2024c). They reduce toxicity or adverse reactions by minimizing drug exposure to systemic circulation. Moreover, they can enhance the solubility or permeability of biofilms, thereby improving pharmacokinetic parameters such as absorption, distribution, metabolism, and clearance of the drugs (Singh et al., 2024).

In the context of peripheral nerve injury repair, local delivery is clearly more advantageous than systemic delivery. Local delivery ensures that a higher drug concentration is achieved directly at the site of injury, improving the chances of effective nerve regeneration. For instance, Wu et al. (2023) used 3D-printing technology to create a nano-assembled hydrogel containing the prodrug 7,8-dihydroxyflavone and found that it showed notable potential in treating long-gap peripheral nerve injuries.

Similarly, local delivery also appears to be the better choice in cases of injury to specific areas of the CNS, such as spinal cord or brain injuries. For example, Xu et al. (2023b) prepared a supramolecular assembly containing p38 inhibitor (SB203580) and insulin-like growth factor 1 for immunomodulatory therapy and neuroprotective regeneration following spinal cord injury through targeted delivery. Jin et al. (2023) prepared an 18β-glycyrrhetinic acid conjugated polymer nanoparticle to promote neuroprotection following the onset of ischemic stroke by intraventricular injection. Certainly, for certain widespread diseases such as Alzheimer’s disease and Parkinson disease, systemic delivery may be better suited to achieving the desired therapeutic outcomes (Han et al., 2020; Leitão et al., 2023; Foltynie et al., 2024; Liu et al., 2024b).

Overall, local delivery is more appropriate for precise treatment of localized injuries, while systemic delivery is better suited for systemic or diffuse diseases. The choice of the delivery method should be determined on the basis of the specific disease, therapeutic goals, and characteristics of the drug.

### Viral delivery systems *versus* non-viral delivery systems

Viral vectors offer notable advantages in nerve regeneration, particularly in terms of gene-delivery efficiency and targeting precision. Viral vectors such as adenoviruses, adeno-associated viruses, and lentiviruses are capable of efficiently transferring genetic material into neural cells, especially neurons and other hard-to-target cells, demonstrating excellent transduction effectiveness (Leibinger et al., 2021; Shortiss et al., 2021; Sydney-Smith et al., 2022; Cong et al., 2025). A very important advantage of viral vectors is their ability to facilitate the integration of these delivered genes into the host genome to allow long-term, stable expression of the gene. This outcome is essential for nerve-regeneration therapies because of the need for sustained gene expression and uninterrupted delivery of NFs or growth factors to facilitate nerve or neuronal repair and regeneration. Specifically, viral vectors can deliver molecules such as NGF and BDNF, which promote neuronal growth, survival, and axonal extension, thereby facilitating the repair and regeneration of nerve damage (Blaszkiewicz et al., 2024; Cong et al., 2025).

However, despite their high efficiency and feasibility in nerve regeneration, viral vectors also have certain limitations. First, viral vectors can trigger immune responses in the host, particularly with repeated use. The immune system may recognize and eliminate these foreign viruses, leading to reduced therapeutic efficacy. The immunogenicity of viral vectors is a major challenge in their use, especially in clinical applications, where patient immune backgrounds and the potential for repeated treatments require careful consideration (Wang et al., 2019). In addition to the numerous biological barriers created by the immune system, the BBB represents another hurdle. Widely recognized methods for bypassing the BBB include direct parenchymal injection, intrathecal injection, intranasal delivery, and intravenous injection of adeno-associated viruses with enhanced BBB permeability (Ye et al., 2024). Furthermore, while certain viral vectors (such as lentiviruses) can integrate their genetic material into the host genome to enable long-term expression, this process may also cause insertional mutations, increasing the risk of carcinogenesis. Additionally, viral vectors have limited delivery capacity, with most viral vectors only capable of delivering relatively small gene sequences. This poses limitations when delivering larger or more complex genetic material in treatments. Therefore, although viral vectors show notable promise in nerve regeneration, their safety concerns and capacity limitations present challenges for their broader application.

Conversely, non-viral vectors have been extensively employed as potential substitutes for viral systems, especially in nerve-regeneration therapy. Non-viral vectors such as liposomes, polymeric nanoparticles, and other nanomaterials encapsulate genetic material through chemical methods to deliver it to target cells through membrane fusion or endocytosis (Lopes et al., 2017; Wang et al., 2022f; Jiang et al., 2024b). The primary benefit of non-viral vectors is their improved safety profile. They also do not elicit immune responses, nor do they integrate into the genome, precluding integration mutations. These features render non-viral vectors particularly attractive in clinical applications where prolonged exposure or repeated dosing is needed (Zhang et al., 2019). Non-viral vectors can also carry much more cargo, allowing the delivery of larger genes or multiple therapeutic molecules. This is vitally important for complex nerve-regeneration therapies, since such therapies frequently involve concurrent delivery of different NFs or genes to enhance nerve repair and regeneration.

Non-viral vectors, however, deliver genes less efficiently than viral vectors (Zhang et al., 2019). Unlike the natural infection capabilities of viral vectors, non-viral vectors require extra modifications through chemical or physical techniques to improve their transfer efficiency. Consequently, they may be less effective than their viral counterparts in particular situations, more specifically in situations requiring high gene transfection. Substantial progress has been made in the development of non-viral vectors in recent years. For example, Lackington et al. (2018) significantly improved the Schwann cell transfection efficiency of non-viral polyethyleneimine plasmid DNA nanoparticles through nanotechnology, and Hsieh et al. (2018) developed a self-healing hydrogel delivery system with high targeting ability and delivery efficiency using optogenetic tools. However, non-viral vectors still face challenges in terms of efficiency and the persistence of gene expression. Since non-viral vectors generally do not integrate into the host genome, the genes they deliver typically only express transiently within the cells, which can limit their effectiveness in nerve regeneration therapies that require long-term gene expression. In this regard, promoting the long-term expression and high-efficiency transfection of non-viral vectors holds great potential and clinical significance.

## Mechanisms of Targeted Delivery of Neuroregenerative Drugs

The nervous system includes the CNS (brain and spinal cord) and PNS. Injuries to these two systems can lead to serious consequences. In particular, CNS injuries can lead to paraplegia or even death (Srihagulang et al., 2022; Hu et al., 2023b). However, the existing methods for treating CNS injuries are quite limited. As mentioned previously, in the context of biomaterial-based drug delivery for CNS regeneration, in addition to accounting for the intrinsic properties of the materials, the challenges posed by biological barriers are another important consideration. In contrast, due to the higher axonal growth capacity of peripheral nerves, treatments for peripheral nerve injuries have been widely studied (Kaplan and Levenberg, 2022). Currently, the clinical gold standard for treating peripheral nerve injuries, especially those involving defects longer than 30 mm, is autologous nerve grafting (Farole and Jamal, 2008; Moore et al., 2009). Nevertheless, autografts face limitations due to donor-site morbidity, suboptimal functional recovery and prognosis at the injury site, and the limited availability of donor tissue. Fortunately, some biomaterials have already undergone clinical translation (He et al., 2015; Saeki et al., 2018; Kusuhara et al., 2019; Rbia et al., 2019). In the development of treatments for nerve defects, certain drug molecules have played an irreplaceable role (**Table 2**).

**Table 2:** Neuroregenerative drugs and their delivery strategies in the past 3 years

Drug	Mechanism	Side effect of systemic application	Material
Nerve growth factors	Reduce neuronal apoptosis and promote nerve regeneration (Kurozumi et al., 2004; Schäbitz et al., 2007); Promote the survival and growth of both sensory and motor (McMahon et al., 1995; Kaplan and Miller, 2000; Airaksinen and Saarma, 2002); Stimulate the proliferation of Schwann cells, enhance the antioxidant capacity of neurons (Yu et al., 1998; Höke et al., 2003)	Low half-life (Tria et al., 1994); High doses cause nerve regeneration disorders (Eggers et al., 2013)	Nanoparticle (Hernando et al., 2022; Gao et al., 2025); Nanotubes (Tian et al., 2023); Nanomotors (Bao et al., 2023); Nanocarriers (Zhang et al., 2025); Hydrogels (Cai et al., 2022; Gao et al., 2022; Xu et al., 2022a; Chen et al., 2024b; Mendes et al., 2024; Ramos Ferrer et al., 2024); Exosomes (Min et al., 2023; Lian et al., 2024); Conduits (Ozcicek et al., 2024; Wan et al., 2025); Films (Wang et al., 2022f); AAV (Petrosyan et al., 2023; Xiao et al., 2023; Cong et al., 2025); Microspheres (Zhang et al., 2022d)
Chondroitinase ABC	Promote nerve cell axon regeneration (Hettiaratchi et al., 2017)	Deactivation within hours at 37°C (Muir et al., 2019)	Copolymers (Kosuri et al., 2022)
Tacrolimus	Bind to FKBP52, regulate neurite growth, and accelerate nerve regeneration	Nephrotoxicity	Nanofibers (Roberton et al., 2023; Daeschler et al., 2025); Hydrogel (Saffari et al., 2021; Wang et al., 2023a); Conduit (Azapagic et al., 2024; Gregory et al., 2024)
Anti-inflammatory drug		Repeated injections and joint infections	Nanoparticles (Li et al., 2023e; Qian et al., 2024; Yang et al., 2024c); Nanotubes (Tian et al., 2023);
NSAIDs	Downregulate proinflammatory factors and alleviate inflammatory response	Fluid retention, allergy, renal dysfunctions (Crofford, 2013; Qindeel et al., 2019)	Nanovesicles (Guo et al., 2024)
Corticosteroids		Gastrointestinal, cardiac, and cerebral toxicity (Buchman, 2001; Gerwin et al., 2006)	Hydrogels (Maxwell et al., 2022; Kong et al., 2023; Chen et al., 2024b; Sun et al., 2024)
Curcumin, nucleic acid drugs		Low bioavailability (Wang et al., 2018)	Conduits (Giannelli et al., 2024)
ASO	Activate the RNase H1 enzyme; Suppress translation or induce degradation of mRNA	Poor permeability across the BBB	Molecule (Barker et al., 2024); Nanodisk (Aly et al., 2023; Caron et al., 2024); Nanoparticles (Byrnes et al., 2023; Ediriweera et al., 2025); Lipid-ligand (Li et al., 2023c); DNA nanostructure (Kim et al., 2024)
siRNA	RNA interference	Degradation in the bloodstream; Renal clearance; Plasma protein binding; Entrapment by the mononuclear phagocyte system; Difficult to penctrate the cell membrane and BBB	Nanovesicles (Jiang et al., 2024b); Nano-Micelles (Yang et al., 2022e); Nanozyme (Xiong et al., 2023); Hydrogels (Gao et al., 2024b); Exosomes (Geng et al., 2023)
miRNA	Facilitate mRNA degradation; Inhibit translation	Sensitive to ribonucleases; Poor cellular internalization.	Nanoparticles (Yang et al., 2022c); Hydrogels (Chen et al., 2023d; Wan et al., 2023); Exosomes (Wang et al., 2022d)
mRNA	Translate into proteins	Sensitive to ribonucleases; Immunogenicity	Nanoparticle (Yu et al., 2023; Gao et al., 2024a); Microparticles (Khalil et al., 2022); Fibers (Puhl et al., 2023); EVs (Gu et al., 2024)
Other drugs		Difficult to transfect into cells	Hydrogel (Qi et al., 2024)

AAV: Adeno-associated virus; ASO: antisense oligonucleotide; BBB: blood‒brain barrier; EV: extracellular vesicle; FKBP52: FK506-binding protein; miRNA: microRNA; mRNA: messenger RNA; NSAID: nonsteroidal anti-inflammatory drug; siRNA: small interfering RNA.

### Neurotrophic factors

The NFs mentioned above are known to exhibit excellent neuroregenerative effects, even though their mechanisms of action may differ. However, these NFs have short half-lives, and high doses of NFs may lead to axonal and Schwann cell encapsulation, which can impair nerve regeneration. Therefore, low-dose continuous administration is the ideal approach for delivery of NFs (Tria et al., 1994; Nkansah et al., 2008; Eggers et al., 2013). In this context, NF-loaded biomaterial-delivery systems have undergone substantial advancements.

Owing to its well-established stability and significant therapeutic effects on nerve regeneration, NGF is currently the most commonly delivered NF. In the field of peripheral nerve repair, Zeng et al. (2021) the nontoxic chemical crosslinker tripolyphosphate in combination with chitosan was used to encapsulate multiple NGF-loaded PLGA microspheres, forming an ionic hydrogel through ionic interactions. This study demonstrated that this hydrogel possesses good biocompatibility and feasibility for nerve tissue repair, providing an effective delivery system for nerve regeneration. Interestingly, Huang et al. (2022) developed a 3D gradient and linearly arranged magnetic PLGA microcapsule system incorporating NGF on the basis of a gelatin/silk hydrogel. This drug delivery system simultaneously provides controllable magnetic properties and topographical cues, thereby accelerating nerve regeneration. Similarly, NGF has demonstrated significant potential for CNS repair. Notably, Gao et al. (2022) developed an anisotropic silk nanofiber gel containing optimized NGF to regulate the differentiation of neurons and astrocytes, thereby promoting scar-free spinal cord repair.

Generally, due to the limitations of using NFs alone for nerve repair and regeneration, NFs used for drug delivery in biomaterials are often combined with similar factors or other substances. In this regard, Lackington et al. (2019) identified the optimal combination doses of NGF and glial cell line-derived neurotrophic factor (GDNF) and developed a delivery method using PLGA microparticles, which demonstrated notable effects in the repair of peripheral nerve defects in rats. Ozcicek et al. (2024) used PCL/PLGA nerve conduits modified with IKVAV peptide in combination with BDNF and NGF for spinal cord repair and regeneration in rats. Pan et al. (2019) used graphene oxide-PLGA hybrid nanofibers for local delivery of BDNF and insulin-like growth factor 1 to repair the spinal cord.

Currently, despite numerous studies focusing on the neuroregenerative effects of NF delivery, further optimization is required to achieve sustained-release profiles that can ensure long-term bioactivity at the injury site. Second, promoting endogenous NGF production through genetic engineering or leveraging the intrinsic conductive properties of materials to achieve therapeutic effects is undoubtedly an excellent option.

### Chondroitinase ABC

Chondroitinase ABC (ChABC) is a bacterial enzyme capable of degrading glial scars formed by the upregulation of chondroitin sulfate proteoglycans following neurological injuries, such as peripheral nerve injury, stroke, traumatic brain injury, and spinal cord injury (Zuo et al., 1998; Zhang et al., 2021b). Studies have shown that ChABC can promote the axonal regeneration of nerve cells and the recovery of nerve function (Bradbury and Carter, 2011; Bosch et al., 2012; Hettiaratchi et al., 2017). However, its thermal instability (losing activity within a few hours at a physiological temperature of 37°C) requires high-dose administration to achieve therapeutic effects (Muir et al., 2019). This approach not only increases the risk of infection but also affects healthy tissues. Therefore, ChABC can be locally delivered to help degrade inhibitory chondroitin sulfate proteoglycans.

For peripheral nerve repair, Donsante et al. (2020) reported that the sustained release of ChABC from nerve conduits significantly promoted axonal regeneration. Notably, compared with sustained release, pulsed release had a markedly detrimental effect on nerve regeneration, possibly due to unstable ChABC concentrations, which may inhibit axonal regrowth. Compared with that of peripheral nerve injuries, the repair of CNS injuries, especially spinal cord injuries, requires a greater focus on inhibiting or reducing scar formation. Therefore, Hettiaratchi et al. (2019) developed a hydrogel for the stable delivery of ChABC to treat CNS damage caused by stroke. Raspa et al. (2021) developed an injectable hydrogel containing ChABC for the treatment of chronic SCI in rats.

### Tacrolimus

Tacrolimus, also known as FK506, is a U.S. Food and Drug Administration (FDA)-approved calcineurin inhibitor that is widely used in clinical practice. Its neuroregenerative effects were first reported in 1994 (Lyons et al., 1994). Currently, the precise mechanisms underlying the neuroregenerative effects of tacrolimus are not fully understood. However, its neurotrophic action is widely recognized to be mediated through the FK506-binding protein (FKBP52) (Steiner et al., 1997; Gold et al., 1999; Daneri-Becerra et al., 2020). This protein forms heterocomplexes with the 90 kDa heat shock protein and its cochaperone p23 within the neuronal nucleus. FKBP52 plays an essential role in directing the growth cones of regenerating neurites, which respond to both attractive and repulsive chemotactic signals (Quintá and Galigniana, 2012). After neuronal injury, this complex is redistributed to the growth cones of regenerating neurites when exposed to tacrolimus *in vitro*, stimulating their accelerated regeneration *in vivo* (Daeschler et al., 2023). Moreover, FK506 promotes nerve regeneration by reducing neuroinflammation and increasing the number of surviving neurons (Kou et al., 2025). Although systemic delivery of tacrolimus may cause side effects such as nephrotoxicity, these often outweigh the expected therapeutic benefits for patients undergoing neurosurgery (Wei et al., 2024). Therefore, the use of biodegradable drug delivery systems to directly target tacrolimus to the nerve repair site could enhance its clinical efficacy while effectively reducing systemic toxicity.

To promote peripheral nerve regeneration, Daeschler et al. (2025) designed a functional nerve sheath capable of the sustained release of tacrolimus for 31 days and demonstrated positive effects on nerves in both *in vivo* and *in vitro* experiments. Additionally, Gregory et al. (2024) used electrospinning technology to fabricate a polycaprolactone scaffold containing tacrolimus and demonstrated its ability to enhance the regeneration of neural stem cells and rat neurons. Currently, studies on FK506-loaded biomaterials for promoting peripheral nerve regeneration are increasingly focused on their biomechanical adaptability and controlled release properties (Wang et al., 2022e, 2023a). Research on FK506-loaded biomaterials for promoting central nerve regeneration is relatively limited. van der Merwe et al. (2017) developed an elastic polymer matrix capable of delivering FK506 epidurally, reducing the systemic tacrolimus blood concentration and off-target toxicity. The application of tacrolimus in biomaterial-based drug delivery systems has demonstrated excellent efficacy in promoting nerve regeneration. Its clinical use should be further advanced for treating postsurgical nerve injuries in nerve repair procedures.

### Anti-inflammatory drugs

Under normal conditions, immune and immune-like glial cells become activated after nerve injury. This leads to the release of pro-inflammatory cytokines such as interleukin (IL)-1, IL-2, IL-6 and tumor necrosis factor-alpha, which is followed by promotion of macrophage recruitment, and finally, the expression of anti-inflammatory factors such as IL-10 and transforming growth factor-β to attenuate the inflammatory response (Austin and Moalem-Taylor, 2010; Rotshenker, 2011). Although this process helps clear cell debris and improve the environment for nerve regeneration, if it persists, it can lead to cell apoptosis, which is detrimental to regeneration and repair (Rock, 2009). The existing anti-inflammatory drugs that are commonly used in biomaterial drug-delivery strategies include curcumin, non-steroidal anti-inflammatory drugs, tetracycline antibiotics, and glucocorticoids.

For example, Sun et al. (2024) designed a keratin–chitosan hydrogel for sustained release of curcumin to promote peripheral nerve regeneration. Wang et al. (2017) developed a hydrogel for controlled release of minocycline (a tetracycline antibiotic that can reduce microglial activation) to promote spinal cord injury repair. However, solely relying on anti-inflammatory treatment is often insufficient for nerve regeneration, especially in cases involving CNS injury and defects. A more effective strategy is the combination of anti-inflammatory drugs with other neuroregenerative agents. In this regard, Zhang et al. (2021a) developed engineered exosomes combining curcumin and NGF for the treatment of spinal cord injury. Adjunctive anti-inflammatory drugs can also be supported by biomaterials that exhibit high efficiency in axonal regeneration, neuronal regeneration and protection, as well as synaptic reconstruction.

### Nucleic acid drugs

**Figure 4:**
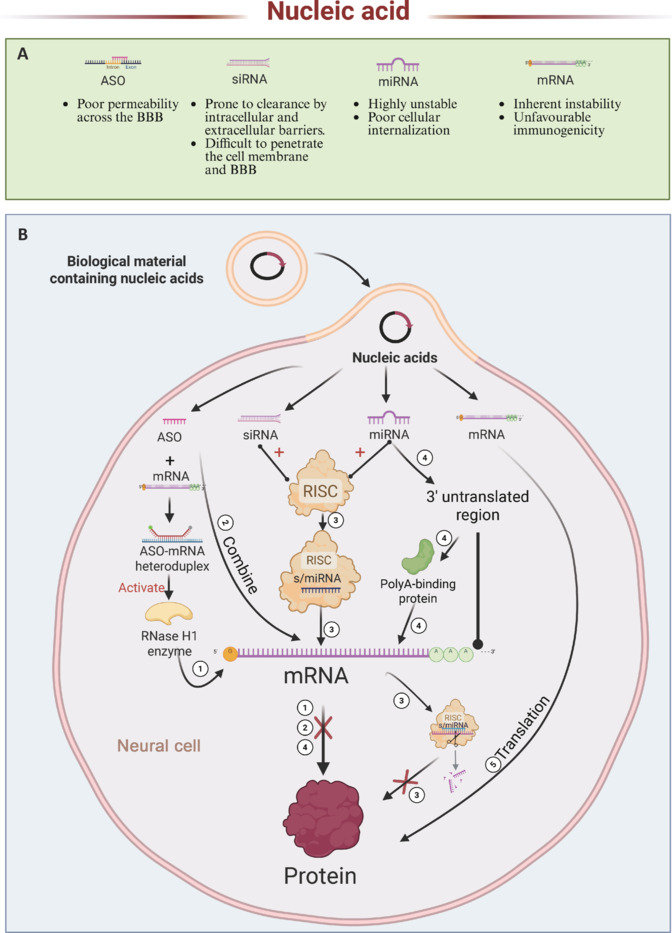
Nucleic acid-based therapeutics: challenges and cellular mechanisms. (A) Challenges of different nucleic acids in therapeutic applications. (B) Cellular mechanisms of nucleic acid processing. Created with BioRender.com. ASO: Antisense oligonucleotide; BBB: blood‒brain barrier; miRNA: microRNA; mRNA: messenger RNA; RISC: RNA-induced silencing complex; siRNA: small interfering RNA.

As a special class of drugs, nucleic acids can regulate the expression of specific genes by influencing the transcription or translation processes and thereby affecting cellular functions. Nucleic acid drugs possess broad therapeutic potential, particularly in the field of nerve regeneration (Anthony, 2022). Nucleic acids are large molecules composed of nucleotides, which consist of bases, ribose, and phosphate groups, and they play a crucial role in the storage and transmission of genetic information. Unlike traditional drugs, nucleic acid-based therapies directly regulate gene expression, allowing precise treatment of diseases. This mechanism allows nucleic acid drugs to demonstrate unique advantages in treating certain complex diseases. A prominent feature of nucleic acid drugs is their ability to directly interfere with the expression of target genes, either upregulating or downregulating specific gene activities. This is particularly important in studies on nerve regeneration, where nucleic acid drugs have attracted widespread attention (Gupta et al., 2021; Brentari et al., 2023). Currently, the main nucleic acids used for drug delivery of neuroregenerative biomaterials include ASOs, siRNA, microRNA (miRNA), and mRNA (**Figure 4**).

#### Antisense oligonucleotides

The ASO is a single-stranded oligonucleotide with a length of 13–25 nucleotides. It forms an ASO-mRNA hybrid with mRNA and activates the RNase H1 enzyme, which specifically cleaves the mRNA, thereby inhibiting the expression of the target gene (Liang et al., 2017; Roberts et al., 2020). ASO can bind to target mRNAs and prevent their normal splicing process, leading to incomplete or incorrect protein synthesis. This mechanism does not rely on RNase H1 but instead interferes with mRNA function through physical blockage (Ramasamy et al., 2022). Many studies have shown the significant effect of ASO on spinal muscular atrophy (Korobeynikov et al., 2022; Tran et al., 2022; Jin and Zhong, 2023; Van Daele et al., 2024). Moreover, notably, in 2016, the FDA approved a drug based on ASO for the treatment of spinal muscular atrophy, Nusinersen (Hoy, 2017). Early studies have shown that inhibiting the activity of sterile-alpha and TIR motif containing 1 (SARM1) can significantly slow the progression of neuronal damage following axonal injury (Coleman and Höke, 2020; Sambashivan and Freeman, 2021). On the basis of these findings, Liu et al. (2023a) locally delivered ASOs into the retina to inhibit the activity of SARM1 and found that they had a significant protective effect on traumatic and glaucomatous optic nerve damage. However, ASOs have poor permeability across the BBB, which reduces the success rate of delivery into the brain and therapeutic efficacy (Yeoh et al., 2024). Current methods of ASO drug delivery focus primarily on the treatment of central nervous system injuries. A key research focus is how to utilize biomaterials to encapsulate ASOs within the brain to enhance their therapeutic effects (Sun et al., 2023; Barker et al., 2024; Caron et al., 2024; Kim et al., 2024).

#### Small interfering RNA

siRNAs are double-stranded RNA molecules that serve as critical tools in gene silencing, primarily by regulating the expression of specific genes through the RNA interference mechanism. Upon entering the cell, the siRNA associates with the RNA-induced silencing complex, a protein complex capable of unwinding the siRNA duplex. The single strand of the siRNA then binds to the complementary sequence of the target mRNA, forming a double-stranded structure. The RNA-induced silencing complex cleaves the target mRNA, thereby rendering it unable to be translated, preventing the production of the corresponding protein (Chiu and Rana, 2003; Moazzam et al., 2024). Interestingly, siRNAs shorter than 15 base pairs are incapable of initiating the RNA interference process, whereas those longer than 30 base pairs may lead to severe complications (Kim et al., 2005). Notably, siRNA therapeutics face various barriers both extracellularly (e.g., degradation in the bloodstream, renal clearance, plasma protein binding, entrapment by the mononuclear phagocyte system, and impermeability of membranes such as the BBB) and intracellularly (Wang et al., 2010; Moazzam et al., 2024). Therefore, research into the chemical modifications of siRNAs and delivery systems is particularly important.

Current research indicates that LNPs, which serve as a delivery platform for Onpattro—the first FDA-approved siRNA drug—have demonstrated significant ability for targeted delivery to neural cells (Khare et al., 2021). On the basis of these findings, in the treatment of peripheral nerves, Komirishetty et al. (2021) reported that delivering Retinoblastoma 1 siRNA alone could enhance axonal regeneration and reinnervation of epidermal axons. Similar to ASOs, research on the therapeutic use of siRNAs for nerve regeneration has focused primarily on the central nervous system. Xiong et al. (2023) developed a neutrophil membrane-coated Mn₃O₄ and IRF-5 siRNA nanoenzyme to promote spinal cord regeneration. Gao et al. (2024b) developed an injectable and photocrosslinkable lipid nanoparticle GelMA hydrogel scaffold for controlled siRNA delivery at the SCI site and reported that it promoted axonal regeneration and myelin regeneration. Furthermore, a key research focus is how siRNAs can be efficiently delivered into the brain—specifically, how to overcome intracellular and extracellular barriers and bypass the BBB to exert their therapeutic effects (Sajid et al., 2020; Zhou et al., 2020). Some researchers argue that systemic administration remains the most ideal approach for siRNA-based therapies for treating neurodegenerative diseases such as Alzheimer’s disease because of its potential for clinical translation (Imran Sajid et al., 2023). The prevailing approaches currently involve intracerebroventricular, intracranial, and intranasal delivery to bypass these barriers (Imran Sajid et al., 2023). Interestingly, Hartl et al. (2023) developed a polyethyleneimine polymer and evaluated its suitability for delivering siRNA across a human-induced pluripotent stem cell-derived BBB model *in vitro*, marking a significant step forward in siRNA transport across the BBB.

#### MicroRNAs

miRNAs are noncoding RNAs that are approximately 20–24 nucleotides in length and play crucial roles in regulating cellular processes. They facilitate the binding of Argonaute proteins to the RNA-induced silencing complex, leading to the cleavage of mRNAs. Additionally, miRNAs target the 3′ untranslated region of mRNAs, promoting their deadenylation. This results in a reduction in interactions with polyA-binding proteins, thereby suppressing mRNA translation or enhancing its degradation, which in turn regulates cellular biological processes such as proliferation, differentiation, and apoptosis (Wu et al., 2006). Notably, miRNAs play indispensable roles in regulating both peripheral and central nerve regeneration, as extensively detailed in comprehensive studies (Zhang et al., 2018; Chang et al., 2024). However, naked miRNAs are highly unstable, are sensitive to ribonucleases and exhibit poor cellular internalization due to their inherent anionic properties. Therefore, more advanced delivery systems are needed to efficiently target and deliver miRNAs, thereby achieving neuroprotection and promoting nerve regeneration (Gupta et al., 2021; Xu et al., 2021).

Considering the need for peripheral nerve regeneration, Wang et al. (2022d) utilized adipose-derived stem cell-derived exosomes to encapsulate miRNA and reinforce the exosomes through low-carbon dioxide stimulation. *In vitro* experiments confirmed that miRNA could successfully enter PC12 cells, and further *in vivo* studies validated its role in sciatic nerve regeneration. This approach not only addresses the challenges of miRNA stability and cellular internalization but also enhances the potential for clinical translation owing to the excellent biocompatibility of exosomes. However, to address central nervous system injuries, researchers often employ injectable miRNA delivery systems to overcome biological barriers. For example, Yang et al. (2022c) developed an injectable nanodelivery system carrying miR-124, which enhances neural stem cell regeneration and thereby improves the treatment of traumatic nerve injuries. Notably, Jiang et al. (2024a) leveraged the ability of exosomes to effectively cross the BBB and screened three targeting peptides, identifying one called RVG29. They then modified exosomes with RVG29 and loaded them with miRNA to achieve therapeutic effects on neurodegenerative diseases in the brain.

#### mRNA

mRNAs can transport genetic information into cells and be translated into proteins and antigenic substances. mRNA therapy promotes nerve regeneration by encoding neurotrophic factors (such as NGF), which induce neuronal survival, axonal growth, and angiogenesis at the site of injury. However, the inherent instability of mRNAs and their unfavorable immunogenicity limit their clinical translation potential (Sahin et al., 2014). However, mRNA delivery vehicles, such as cationic lipids and polymers, can reduce immunogenicity through modifications such as cap analogs and poly(A) tails while preventing mRNA degradation (Karikó et al., 2008; Debus et al., 2010; Williams et al., 2010; Rydzik et al., 2017; Kocmik et al., 2018; Mugridge et al., 2018). Moreover, owing to the negative charge and large molecular size of mRNAs, their efficient delivery into cells remains a major challenge. Research on leveraging specific delivery systems to increase their bioavailability and targeting efficiency has been comprehensively reviewed (Wadhwa et al., 2020).

Currently, studies on local mRNA delivery for promoting peripheral nerve regeneration are relatively abundant. In research on peripheral nerve diseases, Yu et al. (2023) developed an mRNA delivery system encoding NGF, which directly acts on PC12 cells to promote directed axonal growth and remyelination. Similarly, Puhl et al. (2023) used pseudouridine-modified mRNA encoding NT-3 targeted to primary Schwann cells, enhancing their ability to promote DRG neurite outgrowth. In contrast, in CNS regeneration, mRNA delivery faces more complex challenges, such as glial scar formation, inflammatory responses, and an inhibitory microenvironment. mRNA therapy can encode anti-inflammatory factors to reduce inflammation and the accumulation of inhibitory factors, thereby improving the injury microenvironment. By utilizing the antiscar effects of ChABC, Khalil et al. (2022) directly injected an mRNA encoding ChABC into the glial scar and demonstrated its effectiveness in improving spinal cord injury.

### Other drugs

Paclitaxel is a chemotherapeutic drug that has been shown to trigger neuronal regeneration and spinal cord regeneration through the Wnt/β-catenin signaling pathway (Li et al., 2018). However, paclitaxel shows strong peripheral neurotoxicity, which hinders peripheral nerve regeneration (Hsu et al., 2017). Therefore, the use of scaffolds has been shown to be effective for local delivery of paclitaxel to treat spinal cord injury (Fan et al., 2018). Cetuximab, an epidermal growth factor receptor-neutralizing antibody, has been found to promote the neural differentiation of neural progenitor cells, thereby facilitating the repair of spinal cord injury (Li et al., 2013). One study used scaffolds loaded with cetuximab to activate endogenous neuronal differentiation, thereby promoting acute spinal cord repair (Li et al., 2017). Injectable hydrogels encapsulating small molecules such as LDN193189, SB431542, CHIR99021, and P7C3-A20 have been shown to induce endogenous neural stem cells to form neurons and facilitate nerve injury repair (Yang et al., 2021). Fingolimod (FTY720) is a drug used to treat multiple sclerosis. It can promote the proliferation of neural stem cells and shows good neuroprotective effects (Lee et al., 2009). Injectable hydrogels containing FTY720 have also been developed for the treatment of spinal cord injury (Qi et al., 2024).

## Challenges and Strategies for Targeted Delivery of Neuroregenerative Drugs

Peripheral neuropathy, stroke, and spinal cord injury are neurological disorders that severely affect patients’ quality of life and impose substantial societal burdens. Promoting nerve regeneration is a key strategy for treating these conditions, with targeted drug delivery being a crucial component of effective therapies to achieve this outcome. A growing body of research has described various aspects of targeted drug delivery, encompassing a wide range of therapeutic agents. This section of the review focuses on mRNA-based drugs to illustrate the challenges and strategies in targeted drug delivery for nerve regeneration. As an emerging therapeutic tool, mRNA drugs can express specific proteins *in vivo* to regulate the nerve regeneration process. However, the efficacy and safety of these drugs are constrained by various factors. The following paragraphs primarily present the influence of age, sex, pathological conditions, and specific patient populations.

### Effect of age on mRNA neuropharmaceuticals

#### Weakened immune response

With age, the function of the immune system gradually declines, a phenomenon known as immune senescence. Immune senescence manifests as a decline in the number and function of immune cells, such as weakened proliferative capacity of T and B cells and reduced functioning of antigen-presenting cells (Sadighi Akha, 2018). Immune senescence can decrease the immunogenicity of mRNA drugs and weaken the immune system’s ability to recognize and respond to mRNAs, thereby affecting the efficacy of the drugs (Lee et al., 2023). Older adults receiving mRNA vaccines produce lower antibody levels and cellular immune responses than younger adults (Sadighi Akha, 2018). This may be due to the impaired function of antigen-presenting cells in the elderly, which cannot effectively present mRNA to T cells, thereby affecting T cell activation and differentiation (Lee et al., 2023).

#### Changes in drug metabolism

Older adult individuals show reduced function of organs such as the liver and kidney, and the consequent reductions in the activity and expression of drug-metabolizing enzymes cause slower metabolism of drugs in the body (Walmsley et al., 2023). For mRNA drugs, such metabolic changes may lead to increased accumulation of the drug in the body, increasing the risk of adverse reactions (McLachlan and Pont, 2012). One study showed that the metabolic clearance of certain drugs is reduced in the elderly, and the prolonged half-life of these drugs could potentially lead to excessive drug concentrations and trigger toxic reactions (Tajiri and Shimizu, 2013). In addition, changes in body composition and physiologic function in the elderly, such as increased fat content and decreased muscle mass, may also affect drug distribution and metabolism (Farkouh et al., 2020).

#### Decreased nerve-regeneration capacity

Aging leads to reductions in the proliferation and differentiation capacity of neural stem cells and the secretion of NGF as well as changes in the composition and structure of the extracellular matrix, all of which affect nerve-regeneration ability (Li et al., 2024a). Even if mRNA drugs can be successfully delivered to neural tissues, they may not be capable of fully exerting their role in promoting nerve repair due to the deterioration of the nerve regeneration microenvironment (Ryu et al., 2021). The *in vitro* proliferation and differentiation capacity of neural stem cells from the elderly has been shown to be significantly lower than that of neural stem cells from young people (Numakawa and Kajihara, 2023). In addition, the neural tissues of the elderly show increased expression of inflammatory factors and decreased antioxidant capacity, all of which inhibit nerve regeneration (Su et al., 2021).

### Effect of sex on mRNA neuropharmaceuticals

#### Differences in immune response

Women usually show stronger immune responses than men, which may be related to the levels of hormones such as estrogen in women (Gutiérrez-Brito et al., 2024). Estrogen can regulate the functioning of immune cells, enhance their activity and proliferation, and promote the secretion of cytokines (Bachmann et al., 2025). For mRNA drugs, the stronger immune responses in women may lead to faster immune recognition and clearance of drugs, thus affecting the duration and effect of drug action (Demonbreun et al., 2021). The stronger immune responses in women have been suggested to contribute to faster activation of immune mechanisms and therapeutic effects (Sui et al., 2024). However, women may also be at increased risk of immune-related adverse reactions such as allergic reactions and inflammatory reactions (Gutiérrez-Brito et al., 2024).

#### Differences in drug metabolism

Sex differences can also lead to differences in the rate and distribution of drug metabolism. In the liver, a variety of enzymes involved in drug metabolism show sex differences, e.g., some members of the cytochrome P450 enzyme family show different expression and activity patterns in men and women (Sardari et al., 2024). These differences affect the rate of metabolism and peak drug concentrations of mRNA drugs *in vivo*, which, in turn, affects drug efficacy (Rosario et al., 2023). In addition, sex differences affect the distribution of drugs in the body. For example, women have relatively higher adiposity and lower body weight and plasma volume, and these differences in body composition result in higher distribution volumes for lipophilic drugs used in women, which can cause faster onset of action and longer duration of action.

#### Differences in neuroprotective mechanisms

Certain neuroprotective mechanisms differ between males and females. Females possess unique neuroprotective factors such as estrogen, which has neuroprotective effects and can protect nerve cells from injury by regulating their metabolism, limiting anti-oxidative stress, and inhibiting inflammatory responses, among other effects (Yang et al., 2022b). In addition, neural connections and functional networks in the female brain may show greater plasticity and compensatory capacity in response to injury (Paternina-Die et al., 2024). These differences may influence assessments of the effects of mRNA drugs on neural repair as well as evaluations of the safety and tolerability of the drugs (Xia et al., 2024).

### Effects of pathologic states on mRNA neuropharmaceuticals

#### Neurological diseases

Neurological diseases can cause structural and functional damage to the nervous system, resulting in alterations in the function and integrity of the BBB and metabolic and signaling abnormalities in nerve cells (He et al., 2025). Such changes may alter the distribution, metabolism, and/or mechanism of action of mRNA drugs in the nervous system, affecting the patients’ tolerability to mRNA drugs (Bajaj et al., 2024). In patients with Alzheimer’s disease, while the increased permeability of the BBB can facilitate the entry of mRNA drugs in the brain, it can also increase the distribution of drugs in other accessible tissues, thereby affecting the targeting and safety profiles of the drugs (Komal et al., 2025). Moreover, neuroinflammatory responses in patients with neurologic disorders may influence mRNA drug efficacy, and inflammatory factors may hinder the expression and function of mRNA (Kato et al., 2023).

#### Inflammatory state

In the inflammatory state, the body releases a large number of inflammatory factors, such as tumor necrosis factor-alpha and IL-6, which can alter the permeability of the BBB and affect the delivery efficiency of mRNA drugs (Chen et al., 2020). Inflammation can also activate the immune system, and the consequent infiltration of immune cells and increased inflammatory response may affect the stability and safety of mRNA drugs (Dowell et al., 2024). In models of cerebral ischemia-reperfusion injury, inflammatory responses have been shown to cause disruption of the BBB, allowing easier entry of mRNA drugs into the brain but also increasing the risk of drug degradation and adverse reactions (Wang et al., 2022c). In addition, the inflammatory state can cause altered intracellular signaling pathways, which may affect mRNA translation and protein synthesis and thereby affect drug efficacy (Shaw et al., 2025).

### Drug reactions in special populations

#### Immunosuppressed individuals

Immunosuppressed patients, such as organ transplant recipients, patients with human immunodeficiency virus (HIV) infections, and patients with autoimmune diseases, have suppressed immune system function due to long-term use of immunosuppressants or autoimmune system dysfunction (Deepak et al., 2021; Mahrokhian et al., 2022). In immunosuppressed patients, mRNA drugs may elicit a weak immune response, which can affect the efficacy of the drug (von der Schulenburg et al., 2024). Organ transplant recipients receiving mRNA vaccines have been shown to produce significantly lower antibody levels and cellular immune responses than the healthy population (Bader et al., 2023). Moreover, the impaired immune surveillance in immunocompromised patients could render them more vulnerable to infections and tumorigenesis, and the safety of mRNA drugs in these patients should be thoroughly assessed (SeyedAlinaghi et al., 2024). Additionally, the compromised immune system in these patients leaves their body ill-equipped to manage adverse drug responses (e.g., inflammation or systemic reactions), which may be better managed in individuals with a competent immune system.

One of the challenges faced by mRNA drug-delivery systems in immunosuppressed individuals is related to the ability of these systems to enhance therapeutic efficacy while minimizing side effects. In such patients, traditional mRNA drug-delivery strategies may be insufficient. To obtain better outcomes, dose adjustments or combination treatments can be selected. For instance, recipients of organ transplants should receive immunosuppressive agents along with mRNA drugs, and if immunomodulators are added, they can increase the ability of the immune system to respond to mRNA-based therapies (Bachul et al., 2021).

Furthermore, the design and formulation of mRNA delivery materials should be adapted to overcome the specific challenges in immunosuppressed individuals. These populations may require delivery systems capable of better inducing immune responses or more targeted delivery of immune effector cells. Thus, in these populations, the incorporation of adjuvants or the use of advanced delivery technologies such as nanoparticles or LNPs may be employed to augment mRNA therapies, thus ensuring adequate achieved therapeutic effect.

Close monitoring of adverse reactions is essential when mRNA drugs are used in immunosuppressed patients (Canha et al., 2024). Such patients can develop more severe or prolonged reactions than the general population, and prompt adjustment of their treatment plans is necessary to reduce risks. Moreover, the interactions between mRNA drugs and other immunosuppressive treatments should be examined, which may facilitate the design of delivery systems that can work synergistically with these agents.

#### Pregnant women

Pregnant women constitute a separate special population that presents with unique challenges in mRNA drug delivery. Although mRNA-based vaccines have been administered during pregnancy, data on their effects on pregnant women are limited, particularly in the context of other types of mRNA drugs. In comparison with non-pregnant individuals, pregnant women may show a significantly reduced antibody response toward mRNA vaccines early on, but placental and breast milk transfer of antibodies has also been noted, indicating passive immunity for the offspring (Halasa et al., 2022). However, the use of mRNA therapies in this group should be approached with caution, since the major changes in physiology during pregnancy can affect drug metabolism and immune responses.

In this respect, the physiological and hormonal modifications underlying the normal course of pregnancy, including changes such as increased extracellular fluid volume, higher blood volume, and modifications in renal and immune function, require acknowledgment. These alterations may influence the pharmacokinetics and pharmacodynamics of mRNA drugs, potentially complicating the prediction of drug behavior in pregnant women (Munoz et al., 2024). Therefore, the design and delivery of mRNA drugs should account for these elements. Particular care has to be taken to ensure the delivery of mRNA drugs only to the target tissues and minimize any negative impact on the fetus.

The existing research on this topic still does not provide a comprehensive understanding of the long-term effects of mRNA therapies on fetal development. Pregnancy induces unique physiological changes that may alter the pharmacokinetics and pharmacodynamics of mRNA-based drugs in comparison with those in the non-pregnant population. Therefore, optimization of mRNA drug-delivery systems is essential to minimize potential adverse effects on fetal growth and development. This will require either reducing the fetotoxicity of drug-delivery vehicles or refining formulation strategies to develop safer alternatives for use during pregnancy.

The challenge in delivering mRNA drugs to pregnant women is further compounded by the lack of substantial data on the safety of these therapies for both the mother and the fetus. Therefore, careful consideration is required before administering mRNA drugs to pregnant individuals. Continued research into mRNA drug-delivery systems, their safety profiles, and their interactions with the unique physiological changes during pregnancy is essential. More specifically, studies should aim to ensure that these drugs can be delivered effectively without compromising the safety of the mother or the developing fetus.

#### Other special populations

In patients with severe heart disease, respiratory disease, or other chronic diseases, mRNA drugs should be used with more caution due to the presence of complex health conditions and the potential for multiple-organ dysfunction or immune abnormalities (Haq et al., 2022). Drug metabolism and immune responses in these patients may be affected by the disease, increasing the risk of adverse drug reactions (Papadimitriou et al., 2022). Thus, before using mRNA drugs, the patient’s condition, comorbidities, and other factors should be comprehensively evaluated to assess the risks and benefits of the drugs and develop a personalized treatment plan. Subsequently, the patient’s response to the drugs should be carefully monitored, and the therapeutic measures should be adjusted in a timely manner to ensure patient safety and optimize the therapeutic effects.

## Clinical Application Research and Limitations of Targeted Delivery of Neuroregenerative Drugs

Recent research in the field of nerve regeneration has increasingly focused on clinical trials involving targeted drug-delivery systems. Scientists and medical professionals are exploring innovative therapeutic approaches to treat various neurological injuries, including spinal cord injuries, peripheral nerve injuries, and traumatic brain injuries. These conditions often result in severe disabilities, highlighting the importance of effective treatments.

Clinical trials serve as a bridge between laboratory research and practical applications, allowing evaluations of the safety and efficacy of potential therapies. Platforms such as ClinicalTrials.gov and the Chinese Clinical Trial Registry provide valuable insights into ongoing and completed studies, highlighting emerging trends in nerve-regeneration research.

Through the ClinicalTrials.gov platform, many studies have explored the use of *in vitro* wave therapy, electrical stimulation, and ultrasound treatment for nerve regeneration. Additionally, several studies have investigated biological materials, such as the neurospan nerve bridge and neurogen nerve guide developed by auxilium biotechnologies, which aim to repair peripheral nerve defects. One clinical trial (ClinicalTrials.gov ID: NCT06529835) compared these technologies to autologous nerve grafts. Another trial, initiated by the Chinese Academy of Sciences (ClinicalTrials.gov ID: NCT02510365), investigated the use of collagen scaffolds for recovery from SCI.

Moreover, several novel nerve regeneration treatments are undergoing trials. For example, studies are exploring the use of olfactory ensheathing cells for spinal cord injury (ClinicalTrials.gov ID: NCT03933072), gene therapy with Pl-VEGF165 for peripheral nerve damage (ClinicalTrials.gov ID: NCT02352649), and the combination of vitamin B12 and vitamin B3 to promote nerve regeneration and functional recovery in pediatric traumatic brain injury patients (ClinicalTrials.gov ID: NCT05958277). Furthermore, there are trials testing the effect of tacrolimus on peripheral nerve repair (ClinicalTrials.gov ID: NCT00950391).

However, despite the growing body of research, studies on targeted drug delivery for peripheral nerve regeneration remain relatively scarce. A particularly noteworthy study was one initiated by Kunming Tongren Hospital on July 7, 2023, which involved the combination of a stromal vascular fraction with a functional self-assembling peptide nanofiber hydrogel for the treatment of SCI (ClinicalTrials.gov ID: NCT05967325). The stromal vascular fraction is a heterogeneous mixture of cells derived from adipose tissue, including adipose-derived stem cells, endothelial cells, pericytes, T cells, and other immune cells. These cells possess strong self-renewal, proliferation, and differentiation capabilities, allowing them to replace necrotic cells via paracrine and autocrine signaling, synthesize various bioactive factors, and activate cell and vascular regeneration pathways, offering tremendous clinical potential.

Another noteworthy study was published on the Chinese Clinical Trial Registration Center platform and was initiated by the Ninth People’s Hospital of Shanghai Jiao Tong University School of Medicine on August 24, 2020. This clinical trial (Registration No. ChiCTR2000036548) explores the use of bioactive conduits for the sustained release of growth factors to promote peripheral nerve regeneration and functional recovery. The use of bioactive conduits shows significant potential, as the controlled release of growth factors provides targeted and continuous support for nerve repair.

Currently, there are no FDA-approved drugs specifically designed to promote nerve regeneration. However, several FDA-approved therapies have shown potential for nerve repair. Qutenza, a topical treatment, reduces nerve pain through capsaicin and has been used to treat postherpetic neuralgia. Baclofen (Lioresal Intrathecal), an intrathecal delivery medication, effectively alleviates muscle spasticity caused by spinal cord injuries. Stem cell therapies and neurotrophic factor treatments have also made significant progress in multiple clinical trials, particularly in the treatment of spinal cord injuries and neurodegenerative diseases, with promising results. Tacrolimus, an FDA-approved drug, is also used for localized delivery to promote nerve regeneration (Zuo et al., 2021). However, the long-term efficacy and safety of these treatments still require further validation.

Despite some progress, clinical research on targeted drug delivery systems for nerve regeneration still faces numerous challenges. First, many studies have focused primarily on short-term efficacy, whereas nerve regeneration typically requires a longer time frame, meaning that long-term effects and the sustainability of therapeutic outcomes remain pressing issues. Additionally, owing to significant individual patient differences—such as variations in the condition, immune response, and genetic background—treatment effects can vary, making personalized treatment approaches essential. However, existing studies often lack an in-depth exploration of personalized treatment strategies. Moreover, the precision of targeted drug delivery systems remains an unresolved issue. Ensuring that drugs accurately reach damaged areas and maintain their effects is a current technical challenge.

Furthermore, neuroregenerative drugs in clinical applications face safety and side effect concerns. In particular, when stem cell treatments and gene therapies are used, immune responses or other toxic reactions may occur, potentially impacting treatment outcomes and patient health. Finally, ethical issues in nerve regeneration treatments, especially in the stem cell and gene therapy fields, remain critical considerations that must be thoroughly addressed. Therefore, future research must make significant strides in improving efficacy, addressing individual differences, optimizing drug delivery systems, ensuring treatment safety, and resolving ethical concerns.

## Limitations

This review had some limitations, including a restricted scope that may have introduced a bias toward a specific domain while ignoring wider related studies. Another potential source of bias may be the dependence on specific data sources or literature databases, which may have obscured the complete picture. The methodological approach employed in this review, particularly the selection criteria and analytical framework, may have also introduced subjectivity, potentially affecting the comprehensiveness and objectivity of the findings.

## Conclusion

Targeted drug delivery for nerve regeneration is crucial for treating neurological injuries, but current methods show limitations such as adverse reactions, low bioavailability, and rapid drug clearance. Biomaterials such as collagen, hyaluronic acid, chitosan, PLGA, and PEG enable targeted delivery, sustained release, and reduced systemic effects, while advancements in nanotechnology, including exosomes, hydrogels, and nanoparticles, have further enhanced precision. However, clinical translation of these advancements is constrained by concerns regarding material safety, long-term stability, and the complexity of neurodegenerative diseases. Key biological factors, such as Schwann cells, growth factors (NGF, BDNF), and inflammation regulation, play vital roles in nerve repair. Future research should focus on optimizing exosome-based drug delivery, integrating cell therapy with biomaterials, and ensuring precise targeting and safety. The combination of RNA-based therapies (siRNA and mRNA) with nanotechnology holds great promise for nerve injury treatment. With advancements in precision medicine and genomics, personalized drug delivery will enable targeted therapies based on molecular markers and disease progression, enhancing efficacy while minimizing side effects. Strengthening the link between fundamental research and clinical applications is essential for advancing nerve-regeneration therapies. Future efforts should prioritize improving the biocompatibility, degradability, and stability of materials while conducting large-scale clinical trials to ensure safe and effective translation. By leveraging biomaterials, nanotechnology, and innovative therapies, drug-delivery systems for nerve regeneration are expected to significantly improve patient outcomes and quality of life.

**Additional file:** *Open peer review report 1.*

OPEN PEER REVIEW REPORT 1

## Data Availability

*No primary research results, software or code have been included and no new data were generated or analyzed as part of this review*.
