# Supplementary material for: Drug-delivery strategies using biomaterials in the field of nerve regeneration
Source: Neural Regen Res. 2025 Jun 19;21(5):1738–63. doi: 10.4103/NRR.NRR-D-25-00027 (PMC12694642; doi:10.4103/NRR.NRR-D-25-00027)
Supplement: Supplementary file 1 [file NRR-21-1738_Suppl1.pdf]

OPEN PEER REVIEW REPORT 1

Name of journal: Neural Regeneration Research
Manuscript NO: NRR-D-25-00027
Title: Biomaterials and drug delivery strategies for multiple tissues and organs regeneration
Reviewer's Name: Hanin Abdel-Haq
Reviewer's country: Italy

COMMENTS TO AUTHORS

Overall evaluation on article quality:
- The abstract is not well-structured. It must be re-written in order to distinguish a background, a clear and concise description of the issues needed to be addressed, the aims of the review that would address the highlighted issues, methods and results.
- Introduction: Page 8, Line 7, "bodily injuries" is a generic expression while the argument is specific to the nervous system-related injuries.
- Some sections should be shortened, cleaned and in some cases deleted, because same concepts and information are repeated in multiple sections and subsections (for example, Section 4 (4.1 vs 4.2 and 4.3) and section 5), they describe very elementary concepts (section 3) or have been covered by several reviews (sections 3, 5, 4). Indeed;
- The entire section 3 "Biological basis of nerve regeneration" should be shortened as it only describes basic concepts that have been covered in many reviews. Moreover, Section "3.1 Structure and Function of Nerves", unless it is essential to understand the subsequent sections, it is superfluous and unnecessary since it describes very basic notions/concepts. Similarly, section 5 "Functional strategies of biomaterials" besides being superfluous/ describes repeated concepts and information, it has been covered by several reviews.
- Regarding section 4 "Classification and characterization of biomaterials for the preparation of drug delivery systems", the content of subsections 4.2 and 4.3 is almost identical to that described in the subsection 4.1. Besides, these topics are widely covered by many excellent reviews. Therefore, section 4 should be shortened and cleaned.
- Conclusion section is too long and mainly reports a summary of the review. Additionally, in line 19, in the sentence "The field of neuroregenerative drug delivery is an important area of research, especially for the treatment of central and peripheral nerve injuries", especially is not suitable since neuroregenerative is referred to the nervous system.

Timeliness evaluation on article:
The review article covers the most current and relevant literature.

Scope evaluation on article:
The scope of this review article is clear. The authors provided an appropriate overview of the topic.

Direction evaluation on article:
The authors were able to address the main research issues in the present review.

Novelty evaluation on article:

Unfortunately, the review article lacks significant novelty.
